# When do the effects of single‐session interventions persist? Testing the mindset + supportive context hypothesis in a longitudinal randomized trial

**DOI:** 10.1002/jcv2.12191

**Published:** 2023-08-10

**Authors:** Cameron A. Hecht, Samuel D. Gosling, Christopher J. Bryan, Jeremy P. Jamieson, Jared S. Murray, David S. Yeager

**Affiliations:** ^1^ The University of Texas at Austin Austin Texas USA; ^2^ University of Rochester Rochester New York USA

**Keywords:** heterogeneity, long‐term effects, mindsets, single‐session interventions

## Abstract

**Background:**

Single‐session interventions have the potential to address young people's mental health needs at scale, but their effects are heterogeneous. We tested whether the *mindset + supportive context* hypothesis could help explain when intervention effects persist or fade over time. The hypothesis posits that interventions are more effective in environments that support the intervention message. We tested this hypothesis using the synergistic mindsets intervention, a preventative treatment for stress‐related mental health symptoms that helps students appraise stress as a potential asset in the classroom (e.g., increasing oxygenated blood flow) rather than debilitating. In an introductory college course, we examined whether intervention‐consistent messages from instructors sustained changes in appraisals over time, as well as impacts on students' predisposition to try demanding academic tasks that could enhance learning.

**Methods:**

We randomly assigned 1675 students in the course to receive the synergistic mindsets intervention (or a control activity) at the beginning of the semester, and subsequently, to receive intervention‐supportive messages from their instructor (or neutral messages) four times throughout the term. We collected weekly measures of students' appraisals of stress in the course and their predisposition to take on academic challenges. Trial‐registration: OSF.io; DOI: 10.17605/osf.io/fchyn.

**Results:**

A conservative Bayesian analysis indicated that receiving both the intervention and supportive messages led to the greatest increases in positive stress appraisals (0.35 *SD*; 1.00 posterior probability) and challenge‐seeking predisposition (2.33 percentage points; 0.94 posterior probability), averaged over the course of the semester. In addition, intervention effects grew larger throughout the semester when complemented by supportive instructor messages, whereas without these messages, intervention effects shrank somewhat over time.

**Conclusions:**

This study shows, for the first time, that supportive cues in local contexts can be the difference in whether a single‐session intervention's effects fade over time or persist and even amplify.

## Graphical abstract

Single‐session interventions hold promise as scalable treatments for young people, but their effects sometimes persist and sometimes fade out. We found that an established single‐session intervention's effects could be sustained and amplified over time in an introductory college course (*n* = 1675) by providing brief messages from instructors that supported the intervention message. This study provides evidence for a framework that can explain and predict heterogeneity in the effects of single‐session interventions, which will help future researchers and practitioners to shape local environments to sustain the beneficial effects of interventions over time.

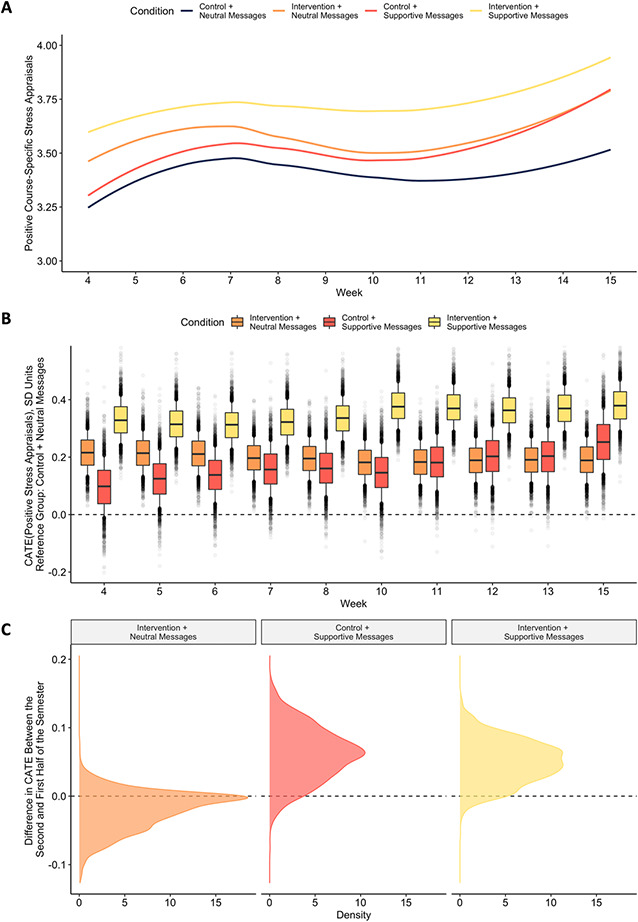

**Key points**

- Single‐session interventions hold promise as scalable treatments for young people's mental health, but their effects sometimes persist and sometimes fade out.
- We found that an established single‐session intervention's effects could be sustained and amplified over time in an introductory college course by providing brief messages from instructors that supported the intervention message.
- This study provides evidence for a framework that can explain and predict heterogeneity in the effects of single‐session interventions, which will help future researchers and practitioners to shape local environments in ways that ensure that these interventions' beneficial effects will be sustained over long periods of time.

## INTRODUCTION

The majority of young Americans do not receive the mental health services they need (Costello et al., 2014; Eisenberg et al., 2011). For instance, only about one third of American college students with mental health needs in a national sample reported receiving treatment in the past year, owing in part to a lack of perceived urgency or skepticism about treatment effectiveness (Eisenberg et al., 2011). One promising approach to help address this problem is the use of single‐session, self‐administered (e.g., online) interventions (see Bloom, 2001; Campbell, 2012; Schleider & Weisz, 2017), which can help to treat or prevent the onset of mental health symptoms among a much broader population of young people. For example, single‐session preventative interventions have been found to reduce heavy drinking (see Samson & Tanner‐Smith, 2015) and increase feelings of hope (Feldman & Dreher, 2012) among college students.

Many single‐session interventions work by shifting beliefs and assumptions that, left unchecked, can negatively affect people over time. For example, a ∼30‐min “synergistic mindsets” intervention aims to correct people's beliefs that stress is always debilitating and should be avoided, which can lead them to disengage from challenging but important stressors and put them at a progressively greater disadvantage over time. The intervention teaches that the stress response can often be *helpful* because it mobilizes the body's resources to take on difficult tasks. This intervention was found to help prevent the onset of stress‐related mental health symptoms among middle‐school, high‐school, and college students across six double‐blind randomized controlled trials (Yeager, Bryan, et al., 2022).

Perhaps the most pressing limitation of single‐session interventions, however, is that their effects are heterogeneous (Schleider & Weisz, 2017). This heterogeneity—when poorly understood—raises questions about the reliability and replicability of these interventions and points to a limited understanding of mechanisms (see Bryan et al., 2021). Furthermore, when these interventions have worked in the short term, their long‐term effects have varied, sometimes growing stronger over time (e.g., as a result of initiating positive behavioral feedback loops; see Hecht et al., 2019; Yeager & Walton, 2011) and sometimes fading out (see Bailey et al., 2017). As a first step to deliver on the promise of single‐session interventions as a scalable way to help prevent growing mental health needs among young people, which have reached record levels (American Psychological Association, 2021; Keeter, 2021), we require a better understanding of where and when these interventions can have lasting impacts on people's beliefs and choices.

Here, we test whether the *mindset + supportive context* hypothesis, which is supported by a growing body of empirical research (e.g., Hecht et al., 2023; Reeves et al., 2020; Yeager, Carroll, et al., 2022; Yeager et al., 2019), can help to address this gap in our current understanding. The mindset + supportive context hypothesis proposes that mindset interventions, which address people's situation‐general belief systems that shape how they interpret broad categories of situations, are more effective in environments that support and are consistent with the proffered mindset (see Hecht et al., 2021; Walton & Yeager, 2020). For example, Yeager, Carroll, et al. (2022) tested a growth mindset intervention in a randomized controlled trial with a nationally representative sample of adolescents. This intervention teaches students the “growth mindset” belief that their intelligence and academic ability can improve with effort, support, and effective strategies. The intervention had a meaningful and significant impact on adolescents' math grades, but only when their teacher also endorsed a growth mindset, presumably creating a classroom environment that supported and reinforced this belief system (see also Hecht et al., 2023).

Despite promising evidence on single‐timepoint outcomes, research has not yet tested the mindset + supportive context hypothesis longitudinally. Therefore, it is still unknown whether supportive contexts help to sustain and reinforce positive initial effects of single‐session interventions. Addressing this question would help us refine our theories of how and when these brief interventions can lead to meaningful long‐term changes, while also informing how effective mental health support could be delivered to a much wider range of young people.

### The synergistic mindsets intervention

The synergistic mindsets intervention is a preventative treatment that has been found to improve stress‐related mental health outcomes among adolescents and young adults (Yeager, Bryan, et al., 2022). The intervention teaches students two complementary mindsets, not as separate ideas, but as coherent parts of one whole. First, the intervention teaches the growth mindset to help students understand stressful academic demands not as problems to be avoided, but instead as opportunities for improvement and growth. Second, the intervention teaches the “stress‐can‐be‐enhancing” mindset: the understanding that the physiological stress response (e.g., increased heart rate) is not always debilitating, but instead can be an enhancing force (e.g., by increasing the flow of oxygenated blood to the brain). This helps students to see the physiological symptoms that tend to accompany academic stressors not as barriers to success, but instead assets that can energize their pursuit of valued goals.

The key to the intervention is that it helps students to reappraise the types of academic stressors that they encounter on a regular basis. By changing these appraisals, the intervention encourages students to proactively engage with challenging coursework rather than avoiding it, which can compound the challenges they face over time and lead them to become overwhelmed.

### The present research

**FIGURE 1:**
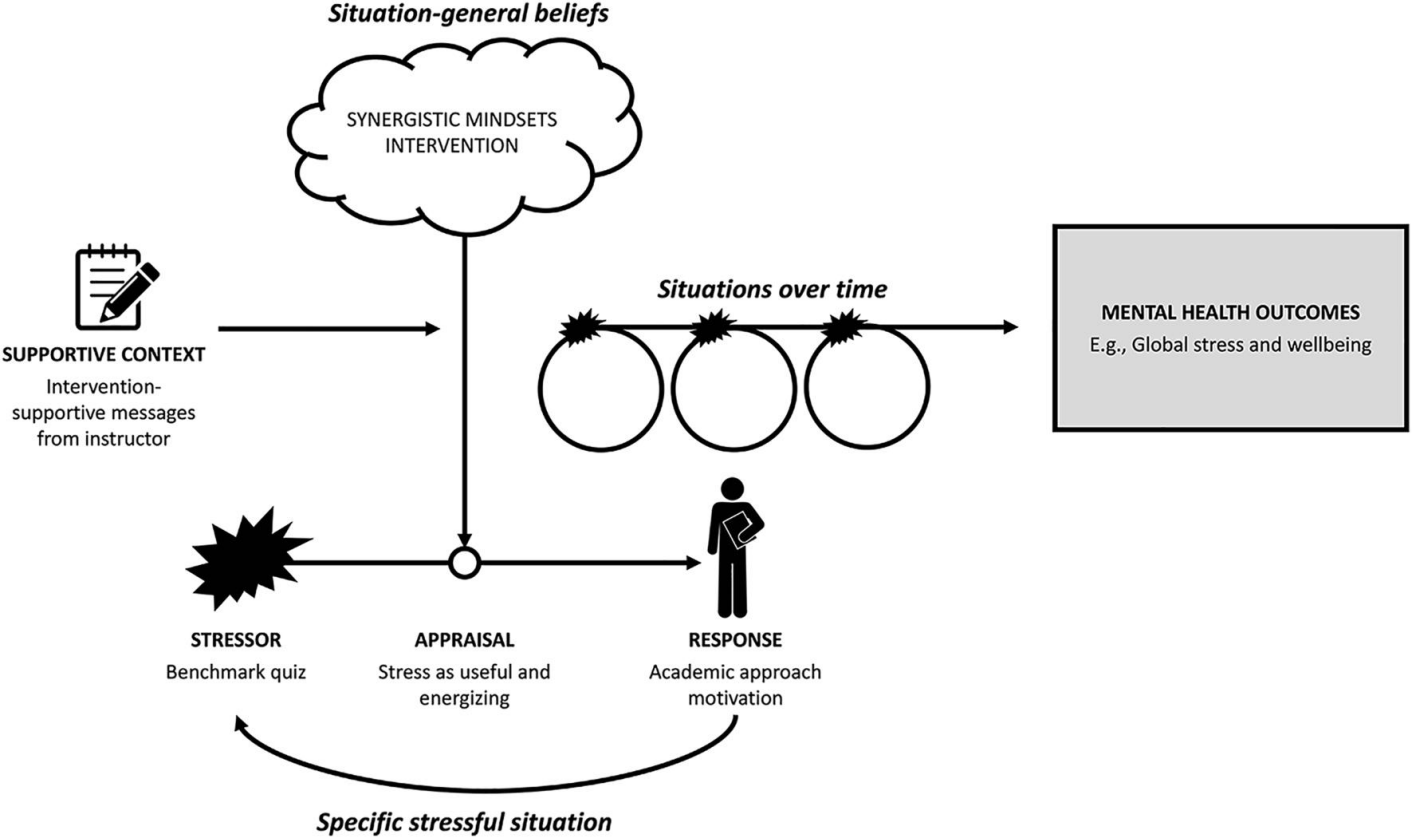
Theory of change. The synergistic mindsets intervention is expected to promote positive appraisals of stress in a specific stressful situation when cues from the context support and reinforce the intervention message. These positive appraisals are, in turn, expected to promote an approach motivated response to the situation (e.g., engaging with demanding academic work, rather than avoiding it). Over time, this approach‐oriented response to stressful situations is expected to result in reduced stress‐related mental health symptoms. As a first step to interrogating this model, this study focuses on stress appraisals and approach‐oriented behavioral intentions, rather than downstream consequences for mental health (depicted in gray).

We randomly assigned undergraduates in an introductory social science course to receive the synergistic mindsets intervention (or control) and to receive regular messages from course instructors that supported the intervention message (or neutral messages). Consistent with previous research (Yeager, Bryan, et al., 2022), we hypothesized that the synergistic mindsets intervention would help college students to appraise stress as a positive and enhancing force within an academic setting, rather than a negative and debilitating force. Further, we reasoned that this change in appraisals might alter students' behavioral intentions, making them more willing to take on (rather than avoid) challenging academic work that could be somewhat stressful but might also promote learning. Critically, we tested the mindset + supportive context hypothesis that these intervention effects would be stronger and more likely to be sustained over time if cues in the academic environment explicitly supported the synergistic mindsets intervention message (see Figure 1).

In this study, we examined how these effects unfolded longitudinally. For instance, we expected that with supportive messages, the synergistic mindsets intervention might sustain students' willingness to take on academic challenges throughout the academic term, whereas this willingness might otherwise decline over the term as academic demands increase. We also tested whether treatment effects were stronger for students who reported higher fixed and stress‐is‐debilitating mindsets pre‐intervention, as has previously been found (Yeager, Bryan, et al., 2022). By testing these hypotheses, the present study adds to our theoretical understanding of when single‐session interventions can initiate meaningful, long‐term changes in young people's beliefs and behavior.

## METHODS

### Participants

**FIGURE 2:**
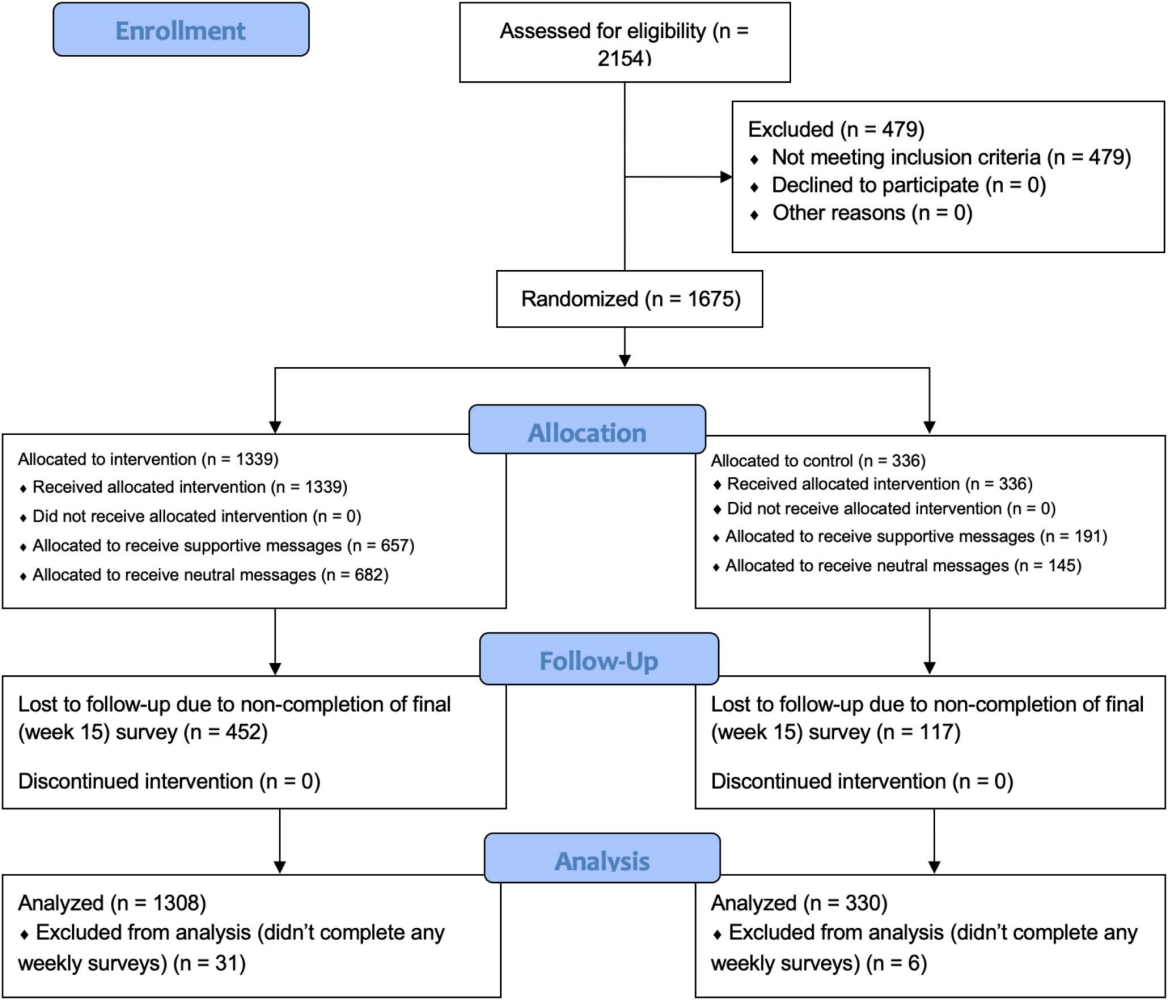
CONSORT diagram for the present study.

The present study was a double‐blind randomized controlled trial conducted in a large, undergraduate introductory social science course at a flagship state university (*N* = 1675; see Figure 2 for CONSORT diagram and Appendix S15 for CONSORT Checklist). This course was administered online, and lectures were provided to students twice per week. The course did not include major “midterm” or “final” exams, but instead short quizzes (called “benchmarks”) that were given during each lecture. Data were collected across two semesters (fall of 2021, *n* = 1075; spring of 2022, *n* = 600). 66% of students were women, 40% were from underrepresented racial/ethnic minority (URM) groups, and 29% were first‐generation (FG) college students (i.e., neither parent had received a 4‐year college degree). See Appendix S2 for additional sample details.

### Procedure

**FIGURE 3:**
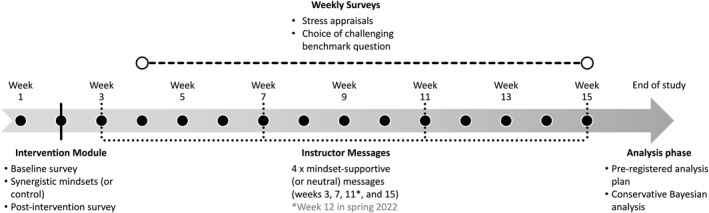
Timeline of study activities in the randomized controlled trial. In week 2, participants completed the intervention module. The module included a baseline survey, the synergistic mindsets intervention or a control activity (depending on condition), and a post‐intervention survey. On weeks 3, 7, 11 (week 12 in spring 2022), and 15, students received a message from their instructor that supported the synergistic mindsets in the context of the course or a neutral message (depending on condition). Between weeks 4 and 15, students completed a survey each Thursday after the course's benchmark quiz that assessed (A) their appraisals of stress in the course, and (B) their choice of a challenging (vs. easy) hypothetical benchmark question. At the end of the study, we analyzed the data following a pre‐registered analysis plan using a conservative Bayesian analysis.

The study procedure is summarized in Figure 3. Students were randomly assigned to one of four conditions in a 2 (Intervention condition: Mindset vs. Control) × 2 (Messages condition: Supportive Messages vs. Neutral Messages) design. Participants had a 75% chance of being randomly assigned to the Mindset intervention condition and a 25% chance of being assigned to the Control condition. This was to ensure that we would have sufficient statistical power to test the effect of receiving supportive messages *among* participants who received the synergistic mindsets intervention, given that the effects of the intervention, as compared to control, were already well established in prior research (Yeager, Bryan, et al., 2022). All participants had a 50% chance of being assigned to the Supportive Messages condition or the Neutral Messages condition.

In the second week of the semester, students completed an intervention module (∼30‐min) that consisted of (a) a baseline survey, (b) an experimental educational activity (the content of which was determined by intervention condition), and (c) a post‐intervention survey. Students in the Mindset condition received the synergistic mindsets intervention and students in the Control condition received a lesson about the brain that did not communicate the synergistic mindsets messages (see Appendix S5 for a description of these activities).

At four times throughout the semester—weeks 3, 7, 11 (week 12 in the spring 2022 semester), and 15—students received a message from their instructors that was embedded in a short (∼5‐min) course activity. In the Supportive Messages condition, this message articulated how the course was designed to support the synergistic mindsets ideas that academic ability can be grown and that the physiological stress response can help students to maximize their learning and performance on academic activities (e.g., tests and quizzes). In particular, the messages focused on how the course included many benchmarks (i.e., short quizzes worth relatively few points), rather than exams, in order to give students many opportunities to practice harnessing their stress response (e.g., increased blood flow to the brain) in performance situations, without suffering major academic consequences as they go through this learning process. In the Neutral Messages condition, the messages described how students could use the benchmarks to gauge their learning progress and identify topics that required further review. See Table 1 for examples of the supportive and neutral messages and Appendix S6 for the complete set. After receiving these messages, students were asked to complete a brief writing exercise prompting them to reflect on the message they had received and how they would approach the upcoming benchmark. This writing activity was intended to facilitate engagement with the instructor's message, while helping students to think about how they could practically implement the message in the course.

**TABLE 1:** Examples of messages provided by instructors that supported the synergistic mindsets intervention message (“supportive messages”) or were neutral with regard to the intervention message (“neutral messages”).

Supportive messages	Neutral messages
Message 1: “Last week, you watched a lecture about how stress isn't always bad and can actually improve your learning and performance. For example, when taking the benchmarks, you may feel your heart rate increase—this is your body providing more oxygenated blood to your brain to help it perform its best.	Message 1: “Throughout this course, one goal will be to check up on our learning and progress. As we will discuss, the benchmarks are one great way to do just that. We hope that as you progress through the topics this semester, you will notice the continuity of ideas and approaches psychologists take to address different research questions, and we also hope you'll see how the topics we study relate to everyday life.
We have designed this class with this fact about the benefits of stress in mind. This is one of the main reasons we give you benchmark quizzes on a regular basis. These benchmarks are timed and intended to be challenging. We have intentionally made the benchmarks frequent but worth a relatively small number of points because this gives you an opportunity to practice channeling your body's stress response in a positive way, without suffering major consequences as you go through this learning process.”	One reason that we use benchmarks in this class, rather than traditional exams, is that they allow you to frequently gauge your progress toward your learning goals. With each benchmark, you can assess how well you have mastered a given topic. In addition, because the benchmarks are cumulative, they can help you apply previous methods and theories we covered to understand new topics. When you don't perform as well as you would like to on a benchmark, this provides you with valuable information—it tells you which topics would be worth some extra time to review.”
Message 2: “As we've discussed previously, one of our goals in this course is to help you learn to take advantage of and channel the human stress response. As a reminder, what we experience as symptoms of stress (e.g., increased heart rate) is your body preparing you to take on a challenge. For example, when you feel your heart rate speed up, this is your body providing more oxygenated blood to your brain to help it perform its best.	Message 2: “As we've discussed previously, one of our goals in this course is to consistently check up on our learning and progress. As a reminder, we hope that you will continue to notice continuity between the ideas and approaches in psychological research over the semester, and also see how topics we study relate to everyday life.
During the previous reflection activity, you thought about ways that you could learn to benefit from your body's stress response by visualizing the positive processes it creates in your body during the benchmarks. Today's reflection activity is a chance to check in on your progress learning to positively channel your stress response so far.”	During the previous reflection activity, you thought about ways that you could use the benchmarks to frequently gauge your progress toward your learning goals. Today's reflection activity is a chance to check in on your progress learning to use the benchmarks in this way.”

*Note*: Four messages were provided to students over the course of the semester in each condition. See Appendix S6 for the full set of messages.

From weeks 4–15, students completed a brief survey after their Thursday benchmark. This survey assessed their appraisals of the stress response in regard to class activities, as well as a measure of students' predisposition to seek academic challenges in the class. We exclude survey data from weeks 9 and 14 because the survey was only administered in one of the two semesters in these weeks and missingness was therefore perfectly correlated with semester. Note that we also collected other measures on brief surveys that were administered multiple times per day each Thursday. We do not report effects on these measures in the main text due to prohibitively high levels of missingness (up to 65% missing), but they are detailed in Appendix S11 and Table S4.

### Measures

Scales and reliabilities are provided below. All items were measured on a six‐point *Strongly disagree—Strongly agree* Likert‐type scale, unless otherwise noted. Correlations and descriptive statistics for these measures are presented in Table 2. Measures of fixed mindset, stress‐is‐debilitating mindset, perceived social stress, and stress appraisals are adapted from Yeager, Bryan, et al. (2022) and predisposition to seek academic challenges is adapted from Rege et al. (2021). Full scales are presented in Appendix S3.

**TABLE 2:** Correlations and descriptive statistics for primary study measures.

Variable	1	2	3	4	5	6	7
1. Baseline fixed mindset	—						
2. Baseline stress‐is‐debilitating mindset	0.21***	—					
3. Baseline perceived social stress	0.18***	0.53***	—				
4. Post‐intervention fixed mindset	0.71***	0.13***	0.12***	—			
5. Post‐intervention stress‐is‐debilitating mindset	0.16***	0.39***	0.26***	0.29***	—		
6. Average stress appraisals	−0.21***	−0.37***	−0.32***	−0.24***	−0.44***	—	
7. Average choice of challenging question	−0.09***	−0.09***	−0.11***	−0.09***	−0.18***	0.31***	—
*N*	1675	1675	1675	1528	1530	1638	1638
*M*	2.62	4.22	3.06	2.34	2.90	3.59	0.35
*SD*	1.09	1.07	0.54	1.11	0.89	0.85	0.39

#### Baseline survey

The baseline survey was completed by the entire sample. Fixed mindset beliefs were measured with three items (e.g., “You have a certain amount of intelligence, and you really can't do much to change it”; *α* = 0.90). Stress‐is‐debilitating mindset beliefs were measured with three items (e.g., “The overall effect of stress on my life is negative”; *α* = 0.83). Perceived social stress was measured with 10 items that inquired about thoughts or feelings over the past 2 weeks, measured on a 5‐point *Never—All the time* Likert‐type scale (e.g., “How often have you felt nervous and stressed?”; *α* = 0.83). Levels of these pre‐intervention variables did not significantly vary by condition (see Appendix S4 and Table S1).

#### Post‐intervention survey (manipulation check)

The post‐intervention survey was completed by 91% of the sample. Fixed mindset beliefs were measured with one item (“Your intelligence is something about you that you can't change very much”). Stress‐is‐debilitating mindset beliefs were measured with four items (e.g., “The effects of stress are bad and I should avoid them”; *α* = 0.88).

#### Weekly surveys

Completion of the weekly surveys varied by week, ranging from 66% to 90%. Stress appraisals were measured with four items (e.g., “I felt like my body's stress responses helped my performance on today's benchmark”; *α* ranged from 0.81 to 0.88 from week to week). Students' predisposition for challenge seeking in the course was measured with a task adapted from a measured used in previous research and validated as a predictor of consequential choices, such as subsequent course taking (Hecht et al., 2023; Rege et al., 2021; Yeager et al., 2019). Students were asked to imagine that their weekly benchmark included two additional questions, that they could decide which one to answer, and that they would receive the same number of points for trying either one. One question was framed as an easy review that they could probably get right without thinking very much, whereas the other was framed as a hard challenge that they would probably answer incorrectly but might learn something new. Our measure of challenge seeking was students' choice of the challenging (vs. easy) question.

### Analysis plan

The analysis plan for this study is preregistered at OSF: https://osf.io/fchyn (see also Appendix S14). See Appendix S1 for details about concordance with and deviations from the preregistered analysis plan. We conducted analyses using multilevel, multi‐arm Bayesian causal forest (BCF) models (Hahn et al., 2020) that tested the effects of condition while nesting individual observations within participants using a random intercept. BCF is a machine‐learning algorithm designed to provide precise estimates of interventions' causal impacts. Its increased precision, relative to traditional frequentist estimates, is due in part to its ability to flexibly incorporate covariates and determine their relationship to the outcome (e.g., nonlinear relationships, interactions) in a way that minimizes bias due to chance imbalances in random assignment and using parameters to avoid overfitting the model to the data.

Beyond these general advantages of BCF, this analytic technique was especially well suited for the present research. Specifically, BCF is ideal for repeated measures designs (due to partial pooling, which borrows information from different timepoints to account for missingness and provide accurate estimates over time), designs with multiple treatment arms that necessitate multiple comparisons (due to conservative prior distributions that shrink estimates toward zero and reduce type I error rates), and to examine research questions concerning moderation (due to conservative prior distributions on the moderator function that minimize the likelihood of identifying spurious interaction patterns) (see Hahn et al., 2020).

For each outcome, BCF estimates posterior distribution of treatment effects that is a function of the data and prior distributions designed to shrink treatment effects and moderation patterns toward zero. We summarize the posterior distribution by reporting its average (i.e., the average treatment effect [ATE] estimate) and the average of the distribution within particular subgroups (i.e., the estimates of conditional ATEs [CATEs]). To test for moderation of treatment effects (i.e., statistical interactions), we subtract the posterior distribution of the treatment effect in one subgroup from another to generate a posterior distribution of the difference in CATEs, which informs whether the magnitude of the effect meaningfully differs between subgroups (i.e., is moderated). In addition to these estimates, we report the interval of the distribution from the 10^th^ to 90^th^ percentile to characterize the variability of the distribution, as well as the proportion of the posterior distribution for each estimate that is greater than zero (which has the intuitive interpretation of the probability that the effect is greater than zero; reported as “pr()”). Following our preregistered standards (see https://osf.io/ncxtm), we do not interpret any effects with less than a 75% posterior probability (i.e., interquartile range of the posterior distribution includes zero) to be meaningful, and we report posterior probabilities above 75% continuously (Gelman, 2016; McShane et al., 2019), with higher probabilities reflecting greater confidence in the effect. Detailed descriptions of the terms in the BCF models are included in Appendix S8. Results from frequentist models (Appendix S10 and Table S3) and raw means and standard deviations by condition (Appendix S13 and Tables S6 and S7) yield substantively similar conclusions as the BCF models.

## RESULTS

### Effects on post‐intervention mindset beliefs (manipulation check)

We first examined whether, in the short‐term (i.e., immediately post‐intervention), the intervention successfully reduced the two complementary sets of beliefs that it targeted: fixed mindset and stress‐is‐debilitating mindset beliefs. The synergistic mindsets intervention reduced fixed mindset beliefs by 0.17 *SD* [−0.23, −0.11], pr(ATE < 0) = 1.00, and stress‐is‐debilitating mindset beliefs by 0.67 *SD* [−0.74, −0.60], pr(ATE < 0) = 1.00. The relatively stronger effects on stress‐is‐debilitating beliefs were likely attributable to the fact that participants endorsed these beliefs more strongly than fixed mindset beliefs at baseline (see Table 2) and there was therefore more room for them to be changed by the intervention. Consistent with this possibility, effects on stress‐is‐debilitating beliefs were stronger for students with relatively high levels of these beliefs at baseline (see Appendix S9 for details on moderation by pre‐intervention mindset beliefs).

### Preliminary check for differential attrition

Differential attrition between experimental conditions can bias the results of experiments that use repeated measures (Deke et al., 2017). We therefore did a preliminary test for differential attrition prior to proceeding with longitudinal analyses on stress appraisals and predisposition for challenge seeking. A detailed description of the differential attrition analysis is provided in Appendix S7 (see also Figure S1 and Table S2). The analysis revealed that there were no condition differences in attrition on the surveys (*p*s > 0.447 for each pairwise comparison to the Control + Neutral Messages group), nor were there any interactions between condition and pre‐intervention stress‐is‐debilitating mindset or fixed mindset (*p*s > 0.122).

### Longitudinal effects on stress appraisals

**FIGURE 4:**
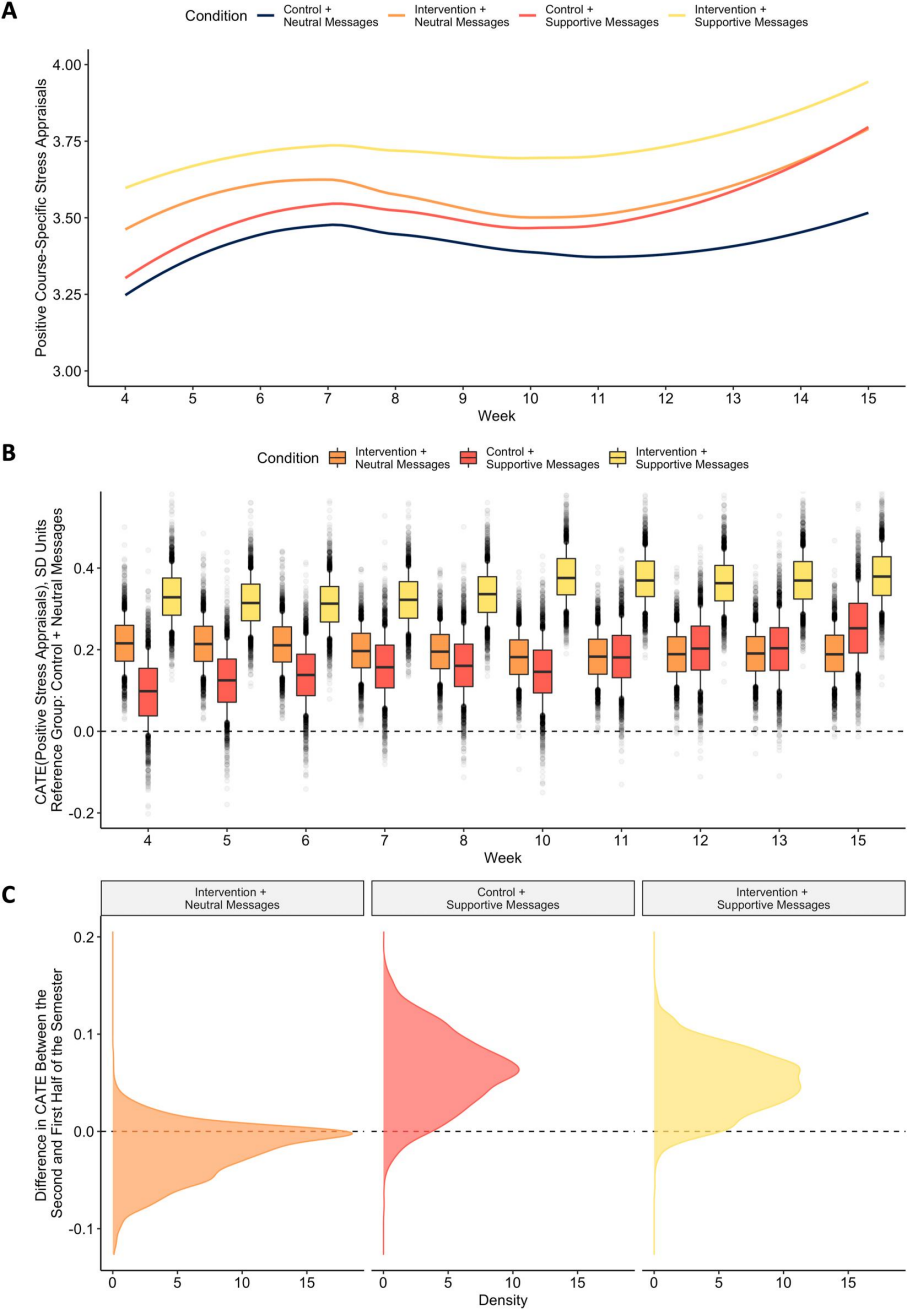
Pattern of effects on course‐specific stress appraisals. Panel A displays estimated stress appraisals as a function of condition and week of the semester using an unconditional model with loess smoothing. Panel B displays the conditional average treatment effect (CATE) for each condition, as compared to the control + neutral messages condition, for each week in the semester at which the outcome was measured, estimated in a Bayesian causal forest model. Boxes represent the interquartile range, whiskers represent the interval from the 10^th^ to 90^th^ percentile of the posterior distribution, and points represent draws from the posterior distribution outside of that range. Panel C displays the posterior distribution of the difference in CATEs for each condition contrast between the second half of the semester (weeks 10–15) and the first half of the semester (weeks 4–8), with a dashed line at zero.

Effects of condition on stress appraisals are displayed over time in Figure 4 and ATEs (averaged across all of the weeks) are reported in Table 3.^1^ Yeager, Bryan, et al. (2022, Study 2) tested the synergistic mindsets intervention in the same class as in the present study and found that the intervention changed students' stress appraisals by 0.18 *SD* 3 weeks post‐intervention. In the present sample, absent supportive instructor messages, we found a similar effect size for the synergistic mindsets intervention 3 weeks post‐intervention (i.e., week 5), CATE_Week5_ = 0.21 *SD* [0.13, 0.29], pr(CATE_Week5_ > 0) = 1.00, as compared to the Control + Neutral Messages condition. However, when students received *both* the intervention and supportive instructor messages, the intervention had an effect of 0.32 *SD* [0.23, 0.40], pr(CATE_Week5_ > 0) = 1.00 (probability of difference between these CATEs = 0.99). The effect of receiving only supportive messages (and the control module) was 0.12 *SD* [0.02, 0.23], pr(CATE_Week5_ > 0) = 0.95.

**TABLE 3:** Average treatment effect estimates for primary outcomes.

Condition contrast (vs. Control + neutral messages)	Positive course‐specific stress appraisals		Choice of challenging question		
	ATE	pr(ATE > 0)	ATE	pr(ATE > 0)	
Intervention + neutral messages	0.20 *SD* [0.12, 0.27]	1.00	1.62 PP [−0.36, 3.67]	0.86	
Control + supportive messages	0.16 *SD* [0.07, 0.25]	0.99	0.05 PP [−1.90, 3.02]	0.59	
Intervention + supportive messages	0.35 *SD* [0.27, 0.42]	1.00	2.33 PP [0.43, 4.41]	0.94	

*Note*: Estimates are derived from a multilevel, multi‐arm Bayesian causal forest model. Average treatment effects (ATEs) are the effect of a given condition, compared to the Control + Neutral Messages condition, averaged across all weeks in which the outcome was measured. In addition to ATEs, we present the interval from the 10th to 90th percentile of the estimate as well as the probability that the ATE is greater than zero.

In addition, whether intervention effects grew stronger or faded over time depended on whether students were provided supportive instructor messages. Absent supportive instructor messages, effects of the synergistic mindsets intervention became somewhat smaller over time (from a maximum of 0.22 *SD* to a minimum of 0.18 *SD*; see Figure 4B). For example, a simple comparison of CATEs between the first half of the semester (weeks 4–8) and the second half of the semester (weeks 10–15) revealed a meaningful, though modest, probability of a reduction in effect size (pr(Difference_CATEs_ > 0) = 0.77). The opposite was the case, however, when the synergistic mindsets intervention was coupled with supportive instructor messages. The effects of the Intervention + Supportive Messages condition grew throughout the semester from a minimum of 0.31 *SD* to a maximum of 0.38 *SD* (see Figure 4B), with a strong probability of an increase in effect size from the first half to the second half of the semester (pr(Difference_CATEs_ > 0) = 0.94). This result is consistent with the mindset + supportive context hypothesis that intervention effects can be sustained and even amplified over time if cues in the context explicitly support the intervention message.

The effect of the Control + Supportive Messages condition also increased over time, from a minimum of 0.10 *SD* to a maximum of 0.25 *SD*, with a strong probability of an increase in effect size from the first half to the second half of the semester (pr(Difference_CATEs_ > 0) = 0.94). The supportive messages may have had only a minor impact on students' stress appraisals early in the semester without the context of the information in the synergistic mindsets intervention, but the effect of these messages may have compounded over the semester as students were continually exposed to them.

Finally, we examined moderation by students' pre‐intervention mindset beliefs and found that the effects of receiving supportive messages—regardless of intervention condition—were somewhat stronger for students who reported higher baseline levels of stress‐is‐debilitating beliefs. See Appendix S9 for additional detail.

### Longitudinal effects on predisposition for challenge seeking

**FIGURE 5:**
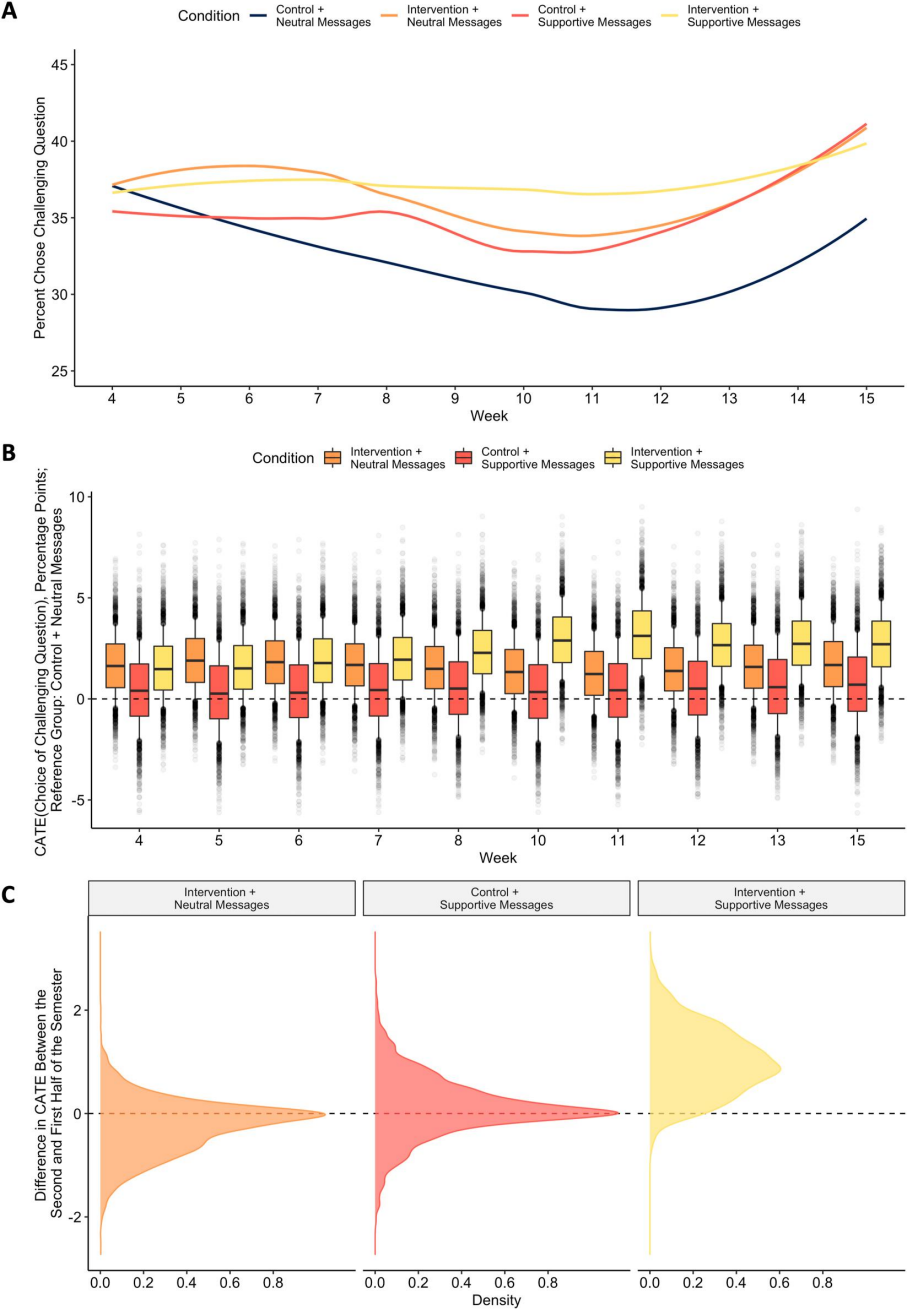
Pattern of effects on choice of a challenging benchmark question. Panel A displays estimated choice of the challenging question as a function of condition and week of the semester using an unconditional model with loess smoothing. Panel B displays the conditional average treatment effect (CATE) for each condition, as compared to the control + neutral messages condition, for each week in the semester at which the outcome was measured, estimated in a Bayesian causal forest model. Boxes represent the interquartile range, whiskers represent the interval from the 10^th^ to 90^th^ percentile of the posterior distribution, and points represent draws from the posterior distribution outside of that range. Panel C displays the posterior distribution of the difference in CATEs for each condition contrast between the second half of the semester (weeks 10–15) and the first half of the semester (weeks 4–8), with a dashed line at zero.

Effects of condition on predisposition for challenge seeking over time are displayed in Figure 5 and ATEs (averaged across all of the weeks) are reported in Table 3. Our challenge‐seeking measure was not collected by Yeager, Bryan, et al. (2022, Study 2), so there is no direct point of comparison with this previous study. However, 3 weeks post‐intervention (i.e., week 5)—the point at which Yeager, Bryan, et al. (2022, Study 2) measured stress appraisals—we found that receiving the synergistic mindsets intervention had similar effects, regardless of whether students received supportive instructor messages. The effect of the Intervention + Neutral Messages condition was 1.93 percentage points [−0.08, 4.02], pr(CATE_Week5_ > 0) = 0.89, and the effect of the Intervention + Supportive Messages condition was 1.58 percentage points [−0.43, 3.70], pr(CATE_Week5_ > 0) = 0.84 (probability of a difference between these CATEs = 0.62). The Control + Supportive Messages condition did not have a meaningful effect (pr(CATE_Week5_ > 0) = 0.55).

However, the effects of receiving the intervention changed over time depending on whether students also received supportive messages from their instructor (Figure 5B). When students did not receive supportive messages, the intervention effect shrank somewhat from a maximum of 1.93 percentage points to a minimum of 1.27 percentage points, though this difference was not meaningful (probability of a reduction in CATEs from the first half to the second half of the semester = 0.68). Conversely, when students *did* receive supportive instructor messages, intervention effects increased over the course of the semester from a minimum of 1.55 percentage points to a maximum of 3.19 percentage points (probability of an increase in CATEs from the first half to the second half of the semester = 0.95). The reason for stronger effects over time seemed to be that, absent intervention and supportive messages, students became less willing to take on academic challenges over time (perhaps due to an increase in stressful academic demands throughout the semester), whereas these rates remained consistently high when students received the synergistic mindsets intervention in addition to supportive messages (see Figure 5A). The difference in effects between the two intervention conditions peaked in week 11, where the effect of the Intervention + Supportive Messages condition was 1.92 percentage points greater than the effect of the Intervention + Neutral Messages condition (pr(Difference_CATEs_ > 0) = 0.95). As with the longitudinal effects on stress appraisals, these findings were consistent with the mindset + supportive context hypothesis that intervention effects can be sustained over time, or even amplified, when implemented in an environment with cues that support the intervention message.

The effect of the Control + Supportive Messages condition did not meaningfully change over time (probability of a difference in CATEs from the first half to the second half of the semester = 0.59). There were no meaningful differences in treatment effects by pre‐intervention mindset beliefs (see Appendix S9 for additional details).

## DISCUSSION

Single‐session interventions can shift young people's beliefs and appraisals of their environments in ways that can benefit their mental health (e.g., Walton & Wilson, 2018; Yeager, Bryan, et al., 2022; see). However, the effects of these interventions are heterogeneous and not consistently sustained over time (see Schleider & Weisz, 2017). Here, we tested whether the *mindset + supportive context* hypothesis could help to explain heterogeneity in treatment effects on students' stress appraisals and associated behaviors using the case of the synergistic mindsets intervention (Yeager, Bryan, et al., 2022). We conducted the first‐ever test of this theory that used repeated measures to look longitudinally at the joint effects of single‐session interventions and supportive contexts. We found that the synergistic mindsets intervention had its strongest effects on students' appraisals of stress in the course and their willingness to take on learning‐oriented academic challenges when they received messages from their instructors that provided clear and context‐specific support for the intervention message.

In addition, whereas intervention effects tended to fade somewhat over time when the context was left unchanged (or stay roughly the same in the case of challenge seeking), introducing supportive instructor messages not only prevented fadeout, but actually led to intervention effects that became larger throughout the semester. This finding is consistent with the possibility that supportive contexts allow the “seed” of an intervention message to take root and create positive feedback loops between belief and behavior that lead to greater improvements over time (see Walton & Yeager, 2020; Yeager & Walton, 2011). The robustness of these findings was bolstered by our use of a pre‐registered study design and analysis plan, as well as a Bayesian analysis that is designed to minimize the likelihood of inflated treatment effect estimates and type I error.

This research also bears on the question of whether dosage, or “boosters,” can amplify the effects of single‐session interventions. Previous research suggests that changing students' mindsets early in an experience (e.g., at the beginning of a course) may be more important than providing the same intervention message multiple times (e.g., Canning et al., 2018; Cook et al., 2012; see Walton & Wilson, 2018). In the present study, however, intervention‐supportive messages that were provided throughout the semester helped to amplify and sustain effects of the synergistic mindsets intervention. These findings suggest that intervention boosters may, in fact, be a promising way to support an initial intervention, but they should not be used to simply repeat the same situation‐general mindset message that students already received. Instead, the goal of these boosters should be to help students see the context as one in which the targeted mindset is welcome and supported.

In addition to these theoretical contributions, the present research has implications for researchers and practitioners who hope to help adolescents and young adults to reappraise academic stressors. In particular, this study suggests that, while teaching students a new way of thinking about the role of stress can be helpful in its own right (e.g., Jamieson et al., 2013; Yeager, Bryan, et al., 2022), we can better support young people by changing the learning environment as well. For example, educators may be better positioned to change students' beliefs about the meaning of stress when they facilitate and reinforce this appraisal of *specific* stressors in a given environment. In the current study, supportive messages focused on short, regular “benchmark” quizzes in the course, and how they provided regular opportunities for students to practice channeling their stress response to perform their best. Instructors who wish to support and reinforce positive appraisals of stress among their students may benefit from identifying (or adding) course components that can be viewed as opportunities to practice using the physiological stress response and communicating that view of these course components to their students.

This work has limitations that should be addressed in future studies that replicate and extend the research. First, completion varied from week to week on the primary outcome measures (from 66% to 90%), making treatment effect estimates less precise in weeks with higher levels of missingness. Ensuring consistently high rates of completion should be a high priority for future work. Second, although the present sample was relatively large, future research should collect a larger sample to ensure high statistical power to detect smaller pairwise differences, such as those between students who received both the intervention and supportive messages and those who received *only* the supportive messages. Finally, the present study was conducted in an introductory social science course at a large, 4‐year university. This research should be replicated in other academic settings in the United States, in different countries and cultures, and with students at different grade levels to examine generalizability and boundary conditions.

In conclusion, the present research bears directly on the question of when and how single‐session interventions can be effective. The research suggests that features of the context can make a substantial difference in whether intervention‐induced changes in thinking fade over time or persist and even grow larger. Attending not only to intervention content but also to the environments in which these interventions are delivered, may enable researchers and practitioners to make real headway in addressing young people's beliefs and assumptions that can affect their mental health.

## AUTHOR CONTRIBUTIONS

**Cameron A. Hecht**: Conceptualization; data curation; formal analysis; investigation; methodology; project administration; resources; visualization; writing—original draft; writing—review and editing. **Samuel D. Gosling**: Resources; writing—review and editing. **Christopher J. Bryan**: Resources; writing—review and editing. **Jeremy P. Jamieson**: Resources; writing—review and editing. **Jared S. Murray**: Software; writing—review and editing. **David S. Yeager**: Conceptualization; formal analysis; funding acquisition; methodology; resources; supervision; writing—review and editing.

## CONFLICT OF INTEREST STATEMENT

The authors have declared that they have no competing or potential conflicts of interest.

## ETHICAL CONSIDERATIONS

This study was approved by UT Austin's Institutional Review Board.

## Supporting information

Supporting Information S1

## Data Availability

The data that support the findings of this study are openly available in OSF at http://doi.org/10.17605/OSF.IO/CFGUX.

## ENDNOTE

^1^
Note that secondary models tested and did not find evidence for moderation of treatment effects by gender, URM, or FG status for stress appraisals or challenge‐seeking predisposition. See Appendix S12 and Table S5.
