# Supplementary material for: When do the effects of single‐session interventions persist? Testing the mindset + supportive context hypothesis in a longitudinal randomized trial
Source: JCPP Adv. 2023 Aug 10;3(4):e12191. doi: 10.1002/jcv2.12191 (PMC10694537; doi:10.1002/jcv2.12191)
Supplement: Supplementary file 1 — Supporting Information S1 [file JCV2-3-e12191-s001.docx]

Supporting Information for “When do the effects of single-session interventions persist? Testing the mindset + supportive context hypothesis in a longitudinal randomized trial”

**Contents**

**Appendix S1. Research Questions and Concordance with Preregistered Analysis Plan**

**Appendix S2.** **Sample Details**

**Appendix S3. Additional Measurement Detail**

**Appendix S4. Experimental Balance on Baseline Characteristics**

**Table S1. Experimental Balance on Pre-Treatment Characteristics**

**Appendix S5. Description of Intervention and Control Modules**

**Appendix S6. Supportive and Neutral Instructor Messages**

**Appendix S7. Tests for Differential Attrition in the Primary Outcome Survey**

**Table S2. Standardized Mean Differences in Completion of the Primary Survey by Week.**

**Figure S1. Percentage of missingness on the primary survey by condition and week. Note: Line width is weighted by number of individuals within a condition.**

**Appendix S8. Description of the Bayesian Causal Forest Models**

**Appendix S9. Moderation by Pre-Intervention Mindsets in Bayesian Causal Forest Models**

**Appendix S10. Multilevel Linear Models for Primary Outcomes**

**Table S3. Fixed Effects from Multilevel Linear Models for Primary Outcomes.**

**Appendix S11. Multilevel Linear Models for Additional Preregistered Outcomes**

**Table S4. Fixed Effects from Multilevel Linear Models for Additional Preregistered Outcomes**

**Appendix S12. Multilevel Linear Models Showing Lack of Demographic Moderation**

**Table S5. Fixed Effects from Multilevel Linear Models Testing Demographic Moderation for Primary Outcomes.**

**Appendix S13. Raw Means and Standard Deviations by Condition**

**Table S6. Raw Means (Standard Deviations) by Intervention Condition for Post-Intervention Manipulation Check Outcomes.**

**Table S7. Raw Means (Standard Deviations) by Condition for Primary Outcomes.**

**Appendix S14. Preregistration File**

**Appendix S15. CONSORT Checklist**

**Overview**

This document provides supplemental information for the research presented in this article.

In the Research Questions and Concordance with Preregistered Analysis Plan section, we present the research questions from our preregistered analysis plans and differences between the reported and planned analyses.

In the Sample Details section, we provide additional information on the sample in this study.

In the Experimental Balance on Baseline Characteristics section, we report tests for balance between the two experimental conditions on baseline variables.

In the Description of Intervention and Control Modules, we present a description of the two experimental modules (reproduced from Yeager et al., 2022).

In the Supportive and Neutral Instructor Messages section, we reproduce the full set of four experimental supportive and neutral instructor messages used in the study.

In the Tests for Differential Attrition in the Primary Outcome Survey section, we provide more detail on how we examined attrition in the study as a function of condition, baseline characteristics, and time.

In the Multilevel Linear Models for Primary Outcomes section, we report findings from multilevel linear models that include the same moderators and covariates as the Bayesian causal forest models.

In the Multilevel Linear Models for Additional Preregistered Outcomes section, we report findings from multilevel linear models on additional preregistered outcomes that were not reported in the main text because of excessive missing data.

In the Multilevel Linear Models Showing Lack of Demographic Moderation, we report findings from multilevel linear models that test gender, race/ethnicity, and first-generation college student status as moderators of condition effects.

In the Raw Means and Standard Deviations by Condition section, we present descriptive statistics to illustrate unconditional differences between experimental conditions.

In the Preregistration File section, we reproduce the full preregistration (also available at <https://osf.io/fchyn>).

In the CONSORT Checklist section, we provide a completed CONSORT checklist for this study.

**Appendix S1. Research Questions and Concordance with Preregistered Analysis Plan**

Below, we list what was planned as an analysis and what analyses were presented in the main text.

Research Questions

*RQ1:* Will students who receive a synergistic mindset intervention and the affordances manipulation show the most positive appraisals of stress?

*RQ2:* Will students who receive a synergistic mindset intervention and the affordances manipulation show the most positive SNS responses (skin conductance and skin temp) during a stressful quiz?

*RQ3:* Will students who receive a synergistic mindset intervention and the affordances manipulation show the most positive daily stress and wellbeing responses?

Planned Analyses

- Our preregistered plan was to use frequentist linear models to test the effects of condition and the moderating influence of time and weekly stress negativity, controlling for covariates, and to use Bayesian causal forest (BCF) models as a robustness check.
- We also preregistered analyses to examine effects on physiological outcomes (i.e., skin conductance events, skin temp events) that were measured within a small subset of the total sample who attended in-person research sessions (RQ2).

Differences between Reported and Planned Analyses

- We reported BCF models as the primary analyses, rather than as a robustness check. The reason is that BCF provides conservative estimates of treatment effects and heterogeneity, reducing the likelihood of type-I error.
- We report estimates from frequentist linear models in Appendix S8. These models yield substantively similar conclusions to those found using BCF.
- The preregistration mistakenly said that RQ1 would be tested using OLS regression, even though the outcome was measured weekly over time and therefore required a nested model structure (similar to that specified for RQ3). Therefore, in the main text we used multilevel BCF models that nest observations within participants, and for comparable frequentist analyses reported in the Supporting Information, we used multilevel linear models (see Appendix S8).
- We had planned to test weekly self-reported stress negativity as a moderator of condition effects. However, we were unable to use this measure (or other measures taken on these surveys, which were administered multiple times per week on Thursdays) due to prohibitively high levels of missingness (as low as 35% completion).
- We included two exploratory moderators that were not preregistered—baseline fixed mindset and baseline stress-is-debilitating mindset—because (a) there was a theoretical rationale to do so (i.e., these are the beliefs that the intervention targets and we suspected that the intervention might reduce these beliefs most among students who most endorsed them at baseline), and (b) because these measures were found to moderate effects of the synergistic mindsets intervention in a previous study (Yeager et al., 2022). Note that BCF’s algorithm is explicitly designed to consider exploratory moderators and is highly robust to identifying spurious patterns of moderation.
- Because the physiological outcomes (RQ2) were collected only for a small subset of the sample and do not relate to our research questions concerning the effects of condition on stress appraisals and related behavioral predispositions (i.e., challenge seeking), they are beyond the scope of the present paper and will be reported in a separate paper.

**Appendix S2.** **Sample Details**

The data for this study came from students across two semesters of an introductory psychology course at a large, public university. The course was provided to students online through videorecorded lectures, and each semester of the course was taught by the same two instructors. There were 1,268 students in the first semester (fall 2021) and 886 students in the second semester (spring 2022). However, 479 students did not begin the intervention (or control) module (i.e., did not see even the first page) and were therefore not included in the analytic sample. This left a final sample of 1,075 students in the fall semester and 600 students in the spring semester (n = 1,675 total).

1,084 students were women, 557 were men, and gender was missing or reported as “other” for 34 students. 549 students were Hispanic/Latinx, 520 students were white, 450 students were Asian or Asian American, 110 students were Black, 11 students were Native Hawaiian or Pacific Islander, 4 students were Native American or Alaska Native, and race was missing or reported as “other” for 31 students. 486 students were first-generation college students (i.e., neither parent had received a four-year degree) and 1,189 students were continuing-generation college students. Models regressing missingness indicators for each demographic variable on experimental condition indicated that missingness did not differ as a function of condition (*p*s > .303).

As described in the main text, participants had a 75% chance of being randomly assigned to the Mindset intervention condition and a 25% chance of being assigned to the Control condition, crossed with a 50% chance of being randomly assigned to the Supportive Messages condition and a 50% chance of being assigned to the Neutral Messages condition. Cell sizes were as follows: Control + Neutral Messages (*n* = 145), Intervention + Neutral Messages (*n* = 682), Control + Supportive Messages (*n* = 191), Intervention + Supportive Messages (*n* = 657).

**Appendix S3. Additional Measurement Detail**

The main text provided a summary of the primary measures in the study. Here we provide a comprehensive report on the measures, including all of the items.

**Baseline survey.** The baseline survey was completed by the entire sample. Fixed mindset beliefs were measured with three items (“You have a certain amount of intelligence, and you really can’t do much to change it,” “You can learn new things, but you can't really change your basic intelligence,” “Your intelligence is something about you that you can't change very much”; *α* = .90). Stress-is-debilitating mindset beliefs were measured with three items (“The overall effect of stress on my life is negative,” “Stress makes it harder for me to learn and grow in school,” “Stress makes it harder to get things done and reach my goals”; *α* = .83). Perceived social stress was measured with ten items that inquired about thoughts or feelings over the past two weeks, measured on a 5-point *Never – All the time* Likert-type scale (“How often have you felt nervous and stressed?”, “How often have you felt able to control the irritations in your life?”, “How often have you been upset because of something that happened unexpectedly?”, “How often have you felt that things were going your way?”, “How often have you been angered because of things that were outside your control?”, “How often have you felt confident about your ability to handle your personal problems?”,“ How often have you felt that you were on top of things?”, “How often have you felt difficulties were piling up so high that you could not overcome them?”, “How often have you felt that you were unable to control the important things in your life?”, “How often have you found that you could not cope with all the things you had to do?”; *α* = .83).

**Post-intervention survey (manipulation check).** The post-intervention survey was completed by 91% of the sample. Fixed mindset beliefs were measured with one item (“Your intelligence is something about you that you can’t change very much”). Stress-is-debilitating mindset beliefs were measured with four items (“The effects of stress are good and I should make use of them,” reversed, “Stress helps me learn and grow,” reversed, “The effects of stress are bad and I should avoid them”, “Stress stops me from learning and growing”; *α* = .88).

**Weekly surveys.** Completion of the weekly surveys varied by week, ranging from 66-90%. Stress appraisals were measured with four items (“I felt like my body's stress responses helped my performance on today's benchmark,” “I felt like my body's stress responses hurt my performance on today's benchmark,” reversed, “Stress makes it harder for me to learn and grow in this psychology class,” reversed, “Stress makes it harder to get things done and reach my goals in this psychology class,” reversed; *α* ranged from .81 - .88 from week to week). Students’ predisposition for challenge seeking in the course was measured with a task adapted from a measured used in previous research and validated as a predictor of consequential choices, such as subsequent course taking (Hecht et al., in press; Rege et al., 2021; Yeager et al., 2019). Students were asked to imagine that their weekly benchmark included two additional questions, that they could decide which one to answer, and that they would receive the same number of points for trying either one—one question was framed as an easy review that they could probably get right without thinking very much, whereas the other was framed as a hard challenge that they would probably answer incorrectly but might learn something new. Our measure of challenge seeking was students’ choice of the challenging (vs. easy) question.

**Appendix S4. Experimental Balance on Baseline Characteristics**

Random assignment produced balance between the four conditions in the 2 (intervention vs. control) × 2 (supportive vs. neutral messages) design in terms of pre-intervention fixed mindset, stress-is-debilitating mindset, and perceived social stress (*p*s > .184). See Table S1.

Table S1. *Experimental Balance on Pre-Treatment Characteristics.*

|  | Control + Neutral Messages | | |  | Intervention + Neutral Messages | | |  | Control + Supportive Messages | | |  | Intervention + Supportive Messages | | |  | Comparison | |
| --- | --- | --- | --- | --- | --- | --- | --- | --- | --- | --- | --- | --- | --- | --- | --- | --- | --- | --- |
| Variable | *M* | *SD* | *N* |  | *M* | *SD* | *N* |  | *M* | *SD* | *N* |  | *M* | *SD* | *N* |  | *F* | *p* |
| Baseline fixed mindset | 2.61 | 1.16 | 145 |  | 2.62 | 1.09 | 682 |  | 2.66 | 1.13 | 191 |  | 2.62 | 1.07 | 657 |  | 0.07 | .975 |
| Baseline stress-is-debilitating mindset | 4.19 | 1.14 | 145 |  | 4.24 | 1.07 | 682 |  | 4.33 | 1.10 | 191 |  | 4.16 | 1.04 | 657 |  | 1.61 | .185 |
| Baseline perceived social stress | 3.02 | 0.54 | 145 |  | 3.07 | 0.54 | 682 |  | 3.12 | 0.58 | 191 |  | 3.04 | 0.53 | 657 |  | 1.46 | .224 |

**Appendix S5. Description of Intervention and Control Modules**

Note: The descriptions below are reproduced directly from Yeager et al. (2022).

**Synergistic mindsets intervention**

The intervention used methods for mindset interventions that are well-established in the literature and have been used successfully in national scale-up studies (Yeager et al., 2019). The intervention first aimed to convey the message that stressful events are controllable and potentially helpful. It did so by targeting negative fixed mindset beliefs, or the belief that intellectual ability is fixed and cannot change, which can lead to the appraisal that negative events are uncontrollable and harmful. In particular, the fixed mindset leads to a pattern of appraisals about effort (that having to try hard or ask for help means you lack ability), about causes of failures (the attribution that failure stems from low ability) and about the desired goal in a setting (the goal of not looking stupid in front of others) (Molden & Dweck, 2006; Yeager & Dweck, 2020). The intervention overcame these negative patterns of appraisals by conveying the growth mindset. The growth mindset promotes the appraisal that difficulties can be controlled and helpful. It argues that most people who became good at something important had to face and overcome struggles, and therefore, your own struggles should not be viewed as signs of deficient abilities but instead should be viewed as part of your path toward important skill development. To justify the controllable and helpful stressor appraisal, the intervention drew on neuroscientific information about the brain’s potential to develop more efficient ('stronger') connections when it faces and overcomes challenges, using the analogy of muscles growing stronger when they are subjected to rigorous exercise (J. M. Aronson et al., 2002).

Second, the intervention targeted the stress-is-debilitating mindset (Jamieson et al., 2018), which is the belief that stress is inherently negative and compromises performance, health and well-being; this mindset leads to the appraisal that a given stressor is uncontrollable and harmful. Counter to the stress-is-debilitating mindset, the intervention developed here introduced the stress-can-be-enhancing mindset (Jamieson et al., 2018), which is the belief that stress can have beneficial effects on performance, health and well-being; this more adaptive belief system leads to the appraisal that stressors can be potentially helpful and controlled. The intervention explained that when people undergo challenges, they inevitably begin to experience stress, which can manifest in a racing heart, sweaty palms or possibly feelings of anxiety or worry. The intervention leads people to perceive those signals as information that the body is preparing to overcome the challenge; for instance, by providing more oxygenated blood to the brain and the muscles (Jamieson et al., 2013). Thus, the stress response is framed as helpful for goal pursuit, not necessarily harmful. The intervention also argued that feelings of anxiety can be a sign that you have chosen a meaningful and ambitious set of goals to work on, and therefore can indicate a positive trajectory, not a negative one.

Notably, these two mindsets were conveyed synergistically, not independently, so that they built on one another. Participants were encouraged to view struggles as potentially positive and worth engaging with, and then they were invited to view inevitable stress coming from this engagement as a part of the body’s natural way to help them overcome the stressor.

These mindset messages were couched within a summary of scientific research on human performance and stress. Participants were not simply informed of these facts, but they were instead invited to engage with them, make them their own and plan how they could use them in the present and future. Participants heard stories from prior participants (older students in this case) who used these ideas to have success in important performance situations, and they also completed open-ended and expressive writing exercises. For instance, participants wrote about a time when they were worried about an upcoming stressor, and then later on they wrote advice for how someone else who might be undergoing a similar experience could use the two mindsets they learned about, which has been called a 'saying-is-believing' writing exercise (E. Aronson, 1999).

**Control group content**

The control group intervention was also an online, self-administered activity lasting around 30 min. It was designed to be relatively indistinguishable from the intervention group by using similar visual layout, fonts, colours and images. The content was predominately from the control condition from a prior national growth mindset experiment (Yeager et al., 2019), which included basic information about the brain and human memory. It also involved open-ended writing activities and stories from older students. However, the control condition did not make any claims about the malleability of intelligence. To this standard content, we added basic information about the body’s stress response system (for example, the sympathetic and parasympathetic nervous system and the HPA axis) to control for the possibility that simply reflecting on stress and stress responses could account for the results. The latter content did not include any evaluations of whether stress responses are good or bad, or controllable or uncontrollable.

**Appendix S6. Supportive and Neutral Instructor Messages**

The activities that included the supportive and neutral instructor messages are reproduced below.

**Activity 1**

Supportive Messages

**Benchmark Reflection Activity #1**

Last week, you watched a lecture about how stress isn’t always bad and can actually improve your learning and performance. For example, when taking the benchmarks, you may feel your heart rate increase – this is your body providing more oxygenated blood to your brain to help it perform its best.

[PAGE BREAK]

**How Benchmarks Support your Learning and Performance**

We have designed this class with this fact about the benefits of stress in mind. This is one of the main reasons we give you benchmark quizzes on a regular basis. These benchmarks are timed and intended to be challenging. We have intentionally made the benchmarks frequent but worth a relatively small number of points because this gives you an opportunity to practice channeling your body’s stress response in a positive way, without suffering major consequences as you go through this learning process.

[PAGE BREAK]

**Reflect for a Moment**

Now, we’d like you to do a little planning for how you’ll approach the benchmarks the rest of this semester. We will have a few more of these reflection activities throughout the semester so that you can check your progress along this journey of learning to positively channel your stress response.

For now, please write a few sentences about what you will say to yourself when you start to feel the symptoms of stress leading up to and during the next benchmark.

Remember: To take advantage of your stress response, you can visualize the positive processes that the stress is in your body. For example, you might Imagine your heart pumping more oxygenated blood to your brain, helping you to think as clearly as possible.

Note: We may anonymously share your response with future students to give them a good example of how to think about the stress and challenge they may experience when taking benchmarks in this class.

Neutral Messages

**Benchmark Reflection Activity #1**

Throughout this course, one goal will be to check up on our learning and progress. As we will discuss, the benchmarks are one great way to do just that. We hope that as you progress through the topics this semester, you will notice the continuity of ideas and approaches psychologists take to address different research questions, and we also hope you’ll see how the topics we study relate to everyday life.

[PAGE BREAK]

**How Benchmarks Help you Gauge Your Progress**

One reason that we use benchmarks in this class, rather than traditional exams, is that they allow you to frequently gauge your progress toward your learning goals. With each benchmark, you can assess how well you have mastered a given topic. In addition, because the benchmarks are cumulative, they can help you apply previous methods and theories we covered to understand new topics. When you don’t perform as well as you would like to on a benchmark, this provides you with valuable information—it tells you which topics would be worth some extra time to review.

[PAGE BREAK]

**Reflect for a Moment**

Now, we’d like you to do a little planning for how you’ll approach the benchmarks the rest of this semester. We will have a few more of these reflection activities throughout the semester so that you can continue to check in on your progress.

For now, please write a few sentences about how you will use the benchmarks to improve your understanding of the material.

Remember: To maximize your learning on the benchmarks, you can relate the topics to other ideas, theories, and methods we have covered so far. You can also take note of the topics that are challenging for you and remember to review them later.

Note: We may anonymously share your response with future students to give them a good example of how to think about the benchmarks.

**Activity 2**

Supportive Messages

**Benchmark Reflection Activity #2**

As we’ve discussed previously, one of our goals in this course is to help you learn to take advantage of and channel the human stress response. As a reminder, what we experience as symptoms of stress (e.g., increased heart rate) is your body preparing you to take on a challenge. For example, when you feel your heart rate speed up, this is your body providing more oxygenated blood to your brain to help it perform its best.

[PAGE BREAK]

**Your Progress So Far**

During the previous reflection activity, you thought about ways that you could learn to benefit from your body’s stress response by visualizing the positive processes it creates in your body during the benchmarks. Today’s reflection activity is a chance to check in on your progress learning to positively channel your stress response so far.

[PAGE BREAK]

**Reflect for a Moment**

Now we’d like you to reflect on your experiences practicing channeling your stress response on the benchmarks so far, and to plan for the remainder of the benchmarks.

**First**, please write a sentence or two about your views on stress and its role in the benchmarks, and how these views may have changed since the beginning of the term.

**Second**, as you did on the previous activity, please write a sentence or two about what you will say to yourself when you start to feel the symptoms of stress leading up to and during the next benchmark.

Here’s what one previous student said. Feel free to draw ideas from this previous student’s response:

“I used to think stress was always bad. But recently, I’ve realized that the symptoms of stress, like my heart racing, is just my body doing what it’s supposed to do: preparing me to do my best.

If I start feeling my heart rate increasing on the next benchmark, I will think about how my body is preparing itself to encounter this challenge to the best of my ability. If I think that I've reached a question which I just can't figure out, I'll think about the neural connections that will be made once I've had the opportunity to go back and review as that will give me the ability to increase my knowledge in the future.”
*This response has been lightly edited for clarity.

Note: We may anonymously share your response with future students to give them a good example of how to think about the stress and challenge they may experience when taking benchmarks in this class.

[PAGE BREAK]

Neutral Messages

**Benchmark Reflection Activity #2**

As we’ve discussed previously, one of our goals in this course is to consistently check up on our learning and progress. As a reminder, we hope that you will continue to notice continuity between the ideas and approaches in psychological research over the semester, and also see how topics we study relate to everyday life.

[PAGE BREAK]

**Your Progress So Far**

During the previous reflection activity, you thought about ways that you could use the benchmarks to frequently gauge your progress toward your learning goals. Today’s reflection activity is a chance to check in on your progress learning to use the benchmarks in this way.

[PAGE BREAK]

**Reflect for a Moment**

Now we’d like you to reflect on your experiences with the benchmarks so far, and to plan for the remainder of the benchmarks.

**First**, please write a sentence or two about how the benchmarks have helped you to track your learning throughout the semester so far.

**Second**, as you did on the previous activity, please write a sentence or two about how you will use the next benchmark (and the benchmarks after that) to continue to improve your understanding.

Here’s what one previous student said. Feel free to draw ideas from this previous student’s response:

"Having a benchmark each week has really helped me to keep up with the material semester. It’s been a good “check-in” to see what I’ve learned and what I still need to work on.

For the next benchmark, I will review past material and try to form connections in order to build my knowledge base. I will also study the previous benchmarks to see where I am getting things wrong.”
*This response has been lightly edited for clarity.

Note: We may anonymously share your response with future students to give them a good example of how to think about the benchmarks.

**Activity 3**

Supportive Messages

**Benchmark Reflection Activity #3**

In the last two benchmark reflection activities, we asked you to think about how you can use the benchmarks as an opportunity to positively channel your stress response. By now, you may have asked yourself: why do we care so much about this?

[PAGE BREAK]

**Why are we Pushing You to Rethink Stress?**

The reason is that, as your instructors, our goal is not to merely help you cope in this class (and in your other classes). Our goal is to help you **thrive**. When instructors try to make material easier for you, they are actually doing you a disservice: they are denying you the opportunity to develop your knowledge and capacity for learning. It is extremely important to us that we do not deny you this opportunity and that, instead, we help push you beyond your previous capabilities and grow your abilities. By helping you to understand the human stress response and learn how to harness it for your own gain, we are pushing you to transform yourself.

[PAGE BREAK]

**Reflect for a Moment**

Now we’d like you to reflect on your experiences practicing channeling your stress response on the benchmarks so far.

**First**, please write 1-2 sentences about how short-term, temporary stressors (like the benchmarks) can help you grow and thrive. For example, you might write about how, by creating moments that feel challenging or uncomfortable, the benchmarks can evoke a stress response, giving you the opportunity to practice channeling it to maximize your performance.

**Second**, as you have done in the previous two reflection activities, please write a sentence or two about what you will say to yourself when you start to feel the symptoms of stress leading up to and during the next benchmark.

Here’s what one previous student said. Feel free to draw ideas from this previous student's response:

“I used to think that if I felt stressed or nervous on a test, that meant I needed to try to calm down. But I realized that there’s actually no need to calm down. If my heart is racing, it just means that more blood is flowing to my brain and it will help me perform better.”

*This response has been lightly edited for clarity.

Remember: To take advantage of your stress response, you can visualize the positive processes that the stress is in your body. For example, you might Imagine your heart pumping more oxygenated blood to your brain, helping you to think as clearly as possible. Or you might imagine your body releasing catecholamines (hormones like adrenaline) that help you think more quickly and solve problems better.

Note: We may anonymously share your response with future students to give them a good example of how to think about the stress and challenge they may experience when taking benchmarks in this class.

Neutral Messages

**Benchmark Reflection Activity #3**

In the last two benchmark reflection activities, we asked you to think about how you can use the benchmarks as an opportunity to gauge your progress learning the material in this class. By now, you may have asked yourself: why do we care so much about this?

[PAGE BREAK]

**Your Progress So Far**

The reason is that, as your instructors, our goal is not to merely help you earn a good grade. Our goal is to help you improve your understanding of the material and retain it long beyond the end of this class. By focusing not only on the benchmark questions you answered correctly, but also attending to the ones you answered incorrectly and correcting your understanding of those concepts, you will remember the concepts and be able to apply them to your life for a long time to come.

[PAGE BREAK]

**Reflect for a Moment**

Now we’d like you to reflect on your experiences gauging your progress on the benchmarks so far

**First**, please write 1-2 sentences about how your approach to learning from the benchmarks can affect your long-term retention and understanding of the material in this class.

**Second**, as you have done in the previous two reflection activities, please write a sentence or two about how you will use the next benchmark (and the benchmarks after that) to continue to improve your understanding.

Here’s what one previous student said. Feel free to draw ideas from this previous student’s response:

"I’ve found it helpful to write down the questions that I get wrong to better understand the topics that I haven’t fully grasped yet. Keeping track of the questions I get right and wrong has helped me to see which topics I’ve really grasped and which ones I still need to work on.”
*This response has been lightly edited for clarity.

Remember: To maximize your learning on the benchmarks, you can relate the topics to other ideas, theories, and methods we have covered so far. You can also take note of the topics that are challenging for you and remember to review them later.

Note: We may anonymously share your response with future students to give them a good example of how to think about the benchmarks.

**Activity 4**

Supportive Messages

**Benchmark Reflection Activity #4**

Over the previous three reflection activities, you have thought about the positive role that the human stress response can have in your learning and performance. As a reminder, the stress response is your body preparing you to take on a challenge (e.g., pumping more oxygenated blood to your brain, releasing hormones like adrenaline that improve your thinking).

[PAGE BREAK]

**Your Journey Harnessing Your Stress Response**

As we approach the end of the semester, we’d like you to take a moment to reflect on your journey this semester learning to harness your stress response to improve your performance. Today’s reflection activity is a chance to think back on your progress so far and to prepare for a strong finish to the semester.

[PAGE BREAK]

**Reflect for a Moment**

Now we’d like you to reflect on your experiences working to harness and channel your stress response on the benchmarks throughout the semester, and to plan for the final few benchmarks.

**First**, please write a sentence or two about how your views on stress and its role in the benchmarks may have changed over the course of term. For example, how do you think your stress response (and your understanding of it) may have helped you perform your best on the benchmarks this semester?

**Second**, as you have done on the previous reflection activities, please write a sentence or two about what you will say to yourself when you start to feel the symptoms of stress leading up to and during the next benchmark.

Here’s what one previous student said. Feel free to draw ideas from this previous student's response:

“Before this class, I thought stress was always a bad thing that kept me from doing my best. But it turns out I had it backwards! My body's stress response can help me to focus all of my energy into the task and hand and perform my best. My heart usually starts racing at the beginning of each benchmark. On the next one, when that happens, I'll picture how my body and brain are getting ready to do their best work.”

*This response has been lightly edited for clarity.

Remember: To take advantage of your stress response, you can visualize the positive processes that the stress is in your body. For example, you might Imagine your heart pumping more oxygenated blood to your brain, helping you to think as clearly as possible. Or you might imagine your body releasing catecholamines (hormones like adrenaline) that help you think more quickly and solve problems better.

Note: We may anonymously share your response with future students to give them a good example of how to think about the stress and challenge they may experience when taking benchmarks in this class.

Neutral Messages

**Benchmark Reflection Activity #4**

In the previous three reflection activities, you have thought about how the benchmarks can be used as an opportunity to gauge your progress learning the material in this class. We hope that you have noticed continuity between the ideas and approaches in psychological research throughout the class so far, and that you have also seen how the topics we study relate to everyday life.

[PAGE BREAK]

**Your Journey Learning from the Benchmarks**

As we approach the end of the semester, we’d like you to take a moment to reflect on your journey this semester learning from this class’s benchmarks. Today’s reflection activity is a chance to think back on your progress so far and to prepare for a strong finish to the semester.

[PAGE BREAK]

**Reflect for a Moment**

Now we’d like you to reflect on your experiences tracking your progress on the benchmarks throughout the semester, and to plan for the final few benchmarks.

**First**, please write a sentence or two about how your understanding of the role of the benchmarks may have changed over the course of the term. For example, how do you think they may have helped you to see connections between different course topics, or to reveal gaps in your understanding?

**Second**, as you have done on the previous reflection activities, please write a sentence or two about how you will use the next benchmark (and the benchmarks after that) to continue to improve your understanding.

Here’s what one previous student said. Feel free to draw ideas from this previous student’s response:

"At the beginning of the semester, I just thought about the benchmarks like any other exam. But I've realized that they actually really help me to check my understanding and see connections between the topics in the course. For the next few benchmarks, I plan to keep checking on what I got wrong so that I can re-study that material and improve my understanding.”

*This response has been lightly edited for clarity.

Remember: To maximize your learning on the benchmarks, you can relate the topics to other ideas, theories, and methods we have covered so far. You can also take note of the topics that are challenging for you and remember to review them later.

Note: We may anonymously share your response with future students to give them a good example of how to think about the benchmarks.

**Appendix S7. Tests for Differential Attrition in the Primary Outcome Survey**

We tested for differential attrition in a model that was identical to the multilevel linear model for the primary outcomes (see Appendix S8), except that the outcome was survey missingness for each week (1 = missing, 0 = not missing). As reported in the main text, there were no overall condition differences in attrition on the surveys (*p*s > .447 for each pairwise comparison to the Control + Neutral Messages group), nor were there any interactions between condition and pre-intervention stress-is-debilitating mindset or fixed mindset (*p*s > .122).

There was, however, some variability in attrition over time. In weeks 4 and 5, the Control + Neutral Messages group had a lower proportion of missingness on the surveys than each of the other conditions (see Table S2 for standardized mean differences in attrition over time and Figure S1 for a visualization). Note, however, that the Control + Neutral Messages condition was the smallest of the four conditions and may have been especially sensitive to small differences in missingness. However, after these first two weeks in which the surveys were measured, there were no significant condition differences in missingness. Finally, note that multilevel BCF analysis—the primary analytic method for this study—uses partial pooling, which borrows information from other timepoints to increase the accuracy of inferences about a given timepoint, making this method especially robust to any patterns of missing data.

Table S2. *Standardized Mean Differences in Completion of the Primary Survey by Week.*

| Week | Intervention + Neutral Messages | Control + Supportive Messages | Intervention + Supportive Messages |
| --- | --- | --- | --- |
| Week 4 | 0.15 | 0.35 | 0.20 |
| Week 5 | 0.22 | 0.23 | 0.22 |
| Week 6 | 0.14 | 0.13 | 0.12 |
| Week 7 | 0.03 | -0.02 | 0.00 |
| Week 8 | -0.02 | -0.09 | -0.01 |
| Week 10 | -0.13 | -0.15 | -0.16 |
| Week 11 | 0.07 | 0.00 | 0.00 |
| Week 12 | 0.01 | 0.05 | -0.03 |
| Week 13 | 0.00 | -0.03 | 0.01 |
| Week 15 | 0.04 | 0.03 | -0.05 |

**
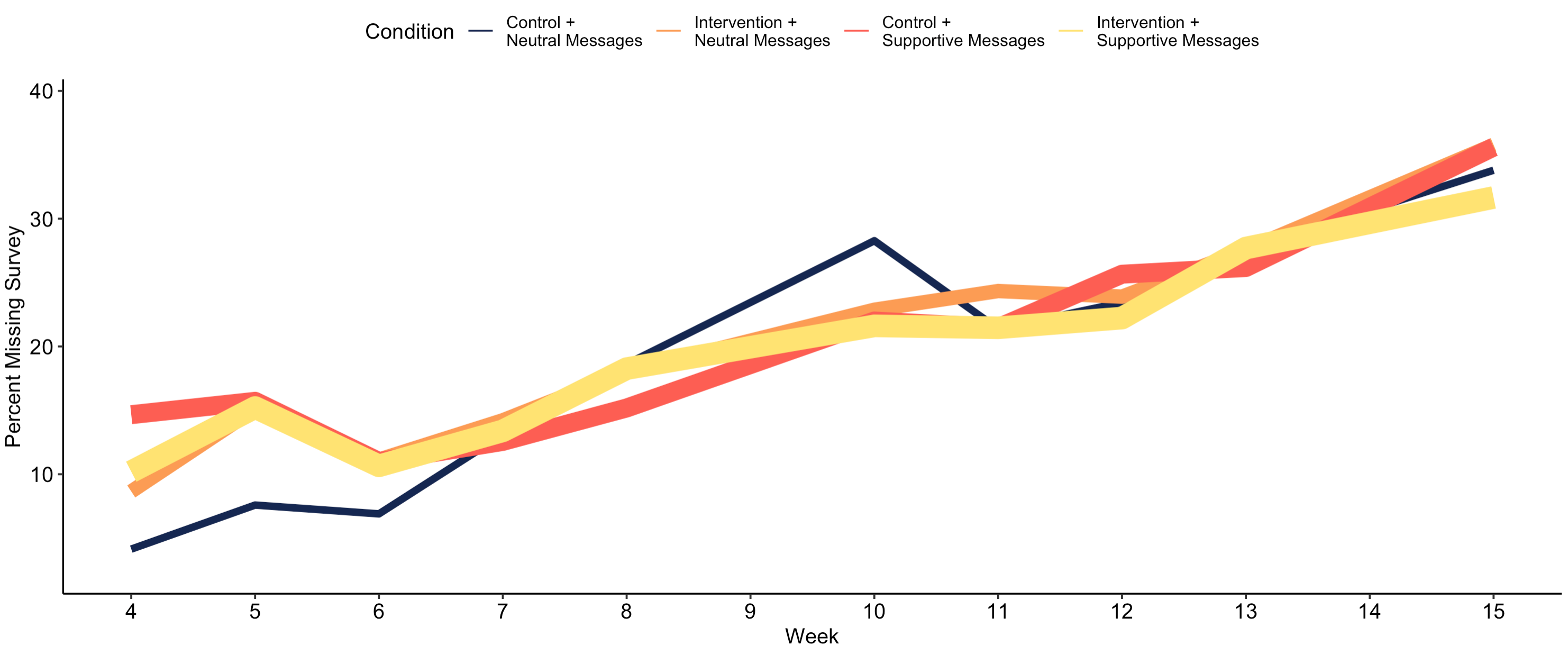
**

*Figure S1.* Percentage of missingness on the primary survey by condition and week. Note: Line width is weighted by number of individuals within a condition.

**Appendix S8. Description of the Bayesian Causal Forest Models**

**Model for post-intervention survey outcomes (manipulation check).** The BCF model for the two outcomes from the post-intervention survey (i.e., fixed mindset and stress-is-debilitating mindset beliefs) tested the effect of the synergistic mindsets intervention and tested pre-intervention fixed mindset and stress-is-debilitating mindset beliefs as potential moderators. This allowed us to examine whether intervention effects may have been larger for students who endorsed high levels of fixed and/or stress-is-debilitating beliefs before receiving the intervention. The model was allowed to incorporate the following covariates: semester, the measures from the pre-intervention survey mentioned earlier, gender, FG status, and URM status. There was no missingness on the pre-intervention survey items. Missingness for demographic variables (2%) were imputed with the mode, and dummy-coded missingness indicators were provided to the model as covariates as well. The model generated a posterior distribution of 2,000 draws after 20,000 burn-in draws, using a thinning interval of 10.

**Model for longitudinal outcomes.** The BCF model for the two outcomes from the weekly surveys was identical to the model for the post-intervention outcomes, with two exceptions. First, because the outcomes were measured repeatedly over time, we used a multilevel BCF model that nested observations within participants by estimating a random intercept. Second, to account for the possibility of heterogeneity in treatment effects over time, we included the week of the semester in which the outcome was measured as a potential within-subjects moderator of treatment effects.

**Appendix S9. Moderation by Pre-Intervention Mindsets in Bayesian Causal Forest Models**

**Post-intervention mindset beliefs (manipulation check).** We compared effects on each of the post-intervention mindset beliefs outcomes for students with relatively high (i.e., ≥ median) levels of pre-intervention fixed and stress-is-debilitating mindsets to those with relatively low (i.e., < median) levels. Effects on fixed mindset beliefs did not vary depending on pre-intervention levels of either type of belief (pr(Difference_CATEs_) < 0.61 for each outcome). However, effects on stress-is-debilitating beliefs were slightly stronger for students with relatively high pre-intervention levels of stress-is-debilitating beliefs, CATE_High_ = -0.72 SD [-0.81, -0.63], pr(CATE_High_ < 0) = 1.00, than for students with relatively low pre-intervention levels of these beliefs, CATE_Low_ = -0.63 SD [-0.72, -0.54], pr(CATE_Low_ < 0) = 1.00 (pr(Difference_CATEs_ > 0) = 0.84). Interestingly, intervention effects on stress-is-debilitating were also slightly stronger for students with relatively low pre-intervention levels of fixed mindset beliefs, CATE_Low_ = -0.71 SD [-0.80, -0.62], pr(CATE_Low_ < 0) = 1.00, than for students with relatively high pre-intervention levels of these beliefs, CATE_High_ = -0.65 SD [-0.73, -0.57], pr(CATE_High_ < 0) = 1.00 (pr(Difference_CATEs_ > 0) = 0.76).

**Stress appraisals.** We examined whether condition effects on stress appraisals varied depending on students’ pre-intervention fixed and stress-is-debilitating mindset beliefs, comparing students with relatively high (i.e., ≥ median) levels of these beliefs those with relatively low (i.e., < median) levels. None of the effects of condition meaningfully varied as a function of students’ pre-intervention fixed mindsets (pr(Difference_CATEs_ > 0) < 0.71 for each condition contrast). However, effects of the two supportive messages conditions were stronger for students who reported higher levels of stress-is-debilitating mindset beliefs at baseline. Specifically, among students with relatively low levels of baseline stress-is-debilitating beliefs, the effect of the Intervention + Supportive Messages condition was 0.33 SD [0.24, 0.41], pr(CATE_Low_ > 0) = 1.00, whereas among students with relatively high levels of these beliefs, the effect was 0.36 SD [0.28, 0.44], pr(CATE_High_ > 0) = 1.00 (pr(Difference_CATEs_ > 0) = 0.79). Similarly, among students with relatively low levels of baseline stress-is-debilitating beliefs, the effect of the Control + Supportive Messages condition was 0.11 SD [0.00, 0.22], pr(CATE_Low_ > 0) = 0.91, whereas among students with relatively high levels of these beliefs, the effect was 0.21 SD [0.12, 0.31], pr(CATE_High_ > 0) = 1.00 (pr(Difference_CATEs_ > 0) = 0.91). The effect of the Intervention + Neutral Messages condition did not meaningfully vary as a function of pre-intervention stress-is-debilitating beliefs (pr(Difference_CATEs_ > 0) = 0.61).

**Predisposition for challenge seeking.** We tested whether condition effects on students’ challenge-seeking predisposition varied as a function of their pre-intervention fixed and stress-is-debilitating mindset beliefs. None of the condition effects varied meaningfully as a function of pre-intervention fixed mindset beliefs or stress-is-debilitating mindset beliefs (pr(Difference_CATEs_ > 0) < 0.70 for each condition contrast and outcome).

**Appendix S10. Multilevel Linear Models for Primary Outcomes**

The findings presented in the main text were estimates from multilevel Bayesian causal forest (BCF) models. Here, we present comparable models using multilevel linear models. The models included condition (dummy-coded contrasts with Control + Neutral Messages as the reference group), week (standardized), the three Condition × Week interactions, baseline fixed mindset (standardized), the three Condition × Fixed Mindset interactions, baseline stress-is-debilitating mindset (standardized), the three Condition × Stress-Is-Debilitating Mindset interactions, baseline perceived social stress, gender (woman = high, man = low; standardized), underrepresented racial/ethnic minority (URM) status (URM = high, majority = low; standardized), and first-generation (FG) college student status (FG = high, continuing-generation = low; standardized). The model included a random intercept for observations within participants, as well as a random slope for week. Fixed effects from these models were consistent with those from the BCF models (see Table S3).

Table S3. *Fixed Effects from Multilevel Linear Models for Primary Outcomes.*

|  | Stress Appraisals | | |  | Choice of Challenging Question | | |
| --- | --- | --- | --- | --- | --- | --- | --- |
| Term | b | SE | t |  | b | SE | t |
| (Intercept) | 3.38 | 0.06 | 52.34 |  | 0.32 | 0.03 | 9.84 |
| Intervention + Neutral Messages | 0.17 | 0.07 | 2.43 |  | 0.04 | 0.04 | 1.11 |
| Control + Supportive Messages | 0.15 | 0.09 | 1.79 |  | 0.02 | 0.04 | 0.36 |
| Intervention + Supportive Messages | 0.33 | 0.07 | 4.55 |  | 0.05 | 0.04 | 1.53 |
| Week | 0.04 | 0.02 | 1.63 |  | -0.02 | 0.01 | -1.62 |
| Intervention + Neutral Messages × Week | -0.02 | 0.03 | -0.67 |  | 0.01 | 0.01 | 0.67 |
| Control + Supportive Messages × Week | 0.05 | 0.03 | 1.41 |  | 0.02 | 0.02 | 0.97 |
| Intervention + Supportive Messages × Week | 0.02 | 0.03 | 0.74 |  | 0.03 | 0.01 | 1.81 |
| Fixed mindset | -0.09 | 0.06 | -1.42 |  | -0.05 | 0.03 | -1.45 |
| Intervention + Neutral Messages × Fixed Mindset | 0.01 | 0.07 | 0.11 |  | 0.02 | 0.03 | 0.68 |
| Control + Supportive Messages × Fixed Mindset | -0.08 | 0.08 | -1.03 |  | 0.01 | 0.04 | 0.27 |
| Intervention + Supportive Messages × Fixed Mindset | -0.05 | 0.07 | -0.66 |  | 0.03 | 0.04 | 0.79 |
| Stress-Is-Debilitating Mindset | -0.37 | 0.06 | -6.04 |  | -0.01 | 0.03 | -0.46 |
| Intervention + Neutral Messages × Stress-Is-Debilitating Mindset | 0.11 | 0.07 | 1.68 |  | -0.01 | 0.03 | -0.19 |
| Control + Supportive Messages × Stress-Is-Debilitating Mindset | 0.29 | 0.08 | 3.54 |  | 0.02 | 0.04 | 0.52 |
| Intervention + Supportive Messages × Stress-Is-Debilitating Mindset | 0.20 | 0.07 | 2.97 |  | 0.01 | 0.04 | 0.34 |
| Perceived Social Stress | -0.14 | 0.02 | -6.33 |  | -0.03 | 0.01 | -2.35 |
| Gender | -0.02 | 0.02 | -0.91 |  | -0.04 | 0.01 | -3.74 |
| URM | -0.04 | 0.02 | -2.02 |  | 0.01 | 0.01 | 1.24 |
| FG | -0.02 | 0.02 | -1.09 |  | 0.01 | 0.01 | 0.72 |

**Appendix S11. Multilevel Linear Models for Additional Preregistered Outcomes**

The remaining preregistered outcomes in the study were measured three times throughout the day each Thursday (at 10am, 1pm, and 7pm). Because the surveys were designed to be brief, each outcome was a single-item measure: positive self-regard (“Overall, how good or bad do you feel about yourself right now?”), perceived social stress (“I feel like I cannot cope with all the things I have to do right now”), and generalized anxiety symptoms (“Right now, how bothered are you by this problem? Feeling nervous, anxious, or on edge”). In addition, one measure was taken only on the 1pm and 7pm surveys: stress negativity (“How negative would you say these experiences were?”, measured after a checklist in which students identified categories of negative events that had happened to them that day, such as events related to academics or relationships). Participants were required to complete at least two of the three surveys per week for at least 10 weeks of the semester in order to receive credit as part of a course assignment. As a result, missingness on any given survey was very high (as high as 65% on a given survey).

The model structure was identical to that used for the primary outcomes (Appendix S10), except that (a) the model also included a random slope for time of day, and (b) the model included the Condition × Week × Time interaction and all corresponding lower-order interactions. Because of high levels of missingness (up to 65%), results on these outcomes should be interpreted with extreme caution, but they are presented in Table S4.

Table S4. *Fixed Effects from Multilevel Linear Models for Additional Preregistered Outcomes*

|  | Positive Self-Regard | | |  | Perceived Social Stress | | |  | Generalized Anxiety Symptoms | | |  | Stress Negativity | | |
| --- | --- | --- | --- | --- | --- | --- | --- | --- | --- | --- | --- | --- | --- | --- | --- |
| Term | b | SE | t |  | b | SE | t |  | b | SE | t |  | b | SE | t |
| (Intercept) | 4.41 | 0.08 | 58.23 |  | 3.09 | 0.07 | 45.31 |  | 2.57 | 0.06 | 44.26 |  | 2.20 | 0.05 | 42.29 |
| Intervention + Neutral Messages | -0.14 | 0.08 | -1.67 |  | 0.10 | 0.08 | 1.34 |  | -0.03 | 0.06 | -0.52 |  | 0.06 | 0.06 | 1.03 |
| Control + Supportive Messages | -0.31 | 0.10 | -3.05 |  | 0.18 | 0.09 | 1.95 |  | 0.15 | 0.08 | 1.97 |  | 0.15 | 0.07 | 2.21 |
| Intervention + Supportive Messages | -0.14 | 0.08 | -1.61 |  | 0.06 | 0.08 | 0.77 |  | -0.06 | 0.06 | -0.96 |  | 0.05 | 0.06 | 0.93 |
| Week | -0.04 | 0.03 | -1.22 |  | -0.01 | 0.03 | -0.49 |  | -0.03 | 0.02 | -1.13 |  | -0.06 | 0.03 | -2.40 |
| Intervention + Neutral Messages × Week | 0.02 | 0.04 | 0.56 |  | -0.01 | 0.03 | -0.34 |  | -0.02 | 0.03 | -0.63 |  | 0.05 | 0.03 | 1.65 |
| Control + Supportive Messages × Week | -0.02 | 0.04 | -0.53 |  | 0.01 | 0.04 | 0.18 |  | -0.02 | 0.03 | -0.52 |  | 0.07 | 0.03 | 1.99 |
| Intervention + Supportive Messages × Week | 0.00 | 0.04 | 0.13 |  | 0.00 | 0.03 | -0.14 |  | 0.01 | 0.03 | 0.24 |  | 0.06 | 0.03 | 2.00 |
| Time | -0.07 | 0.03 | -2.48 |  | -0.02 | 0.02 | -0.92 |  | -0.02 | 0.02 | -0.89 |  | 0.00 | 0.02 | -0.20 |
| Intervention + Neutral Messages × Time | 0.01 | 0.03 | 0.27 |  | 0.00 | 0.02 | -0.11 |  | 0.01 | 0.02 | 0.32 |  | 0.01 | 0.02 | 0.44 |
| Control + Supportive Messages × Time | 0.03 | 0.04 | 0.81 |  | 0.00 | 0.03 | -0.02 |  | 0.00 | 0.02 | -0.07 |  | 0.02 | 0.03 | 0.55 |
| Intervention + Supportive Messages × Time | 0.02 | 0.03 | 0.56 |  | -0.02 | 0.02 | -0.82 |  | 0.00 | 0.02 | 0.05 |  | 0.00 | 0.02 | 0.13 |
| Week × Time | 0.04 | 0.02 | 1.85 |  | 0.01 | 0.02 | 0.44 |  | -0.01 | 0.02 | -0.62 |  | -0.04 | 0.02 | -2.09 |
| Intervention + Neutral Messages × Week × Time | 0.01 | 0.03 | 0.35 |  | -0.02 | 0.02 | -0.82 |  | 0.01 | 0.02 | 0.37 |  | 0.03 | 0.02 | 1.40 |
| Control + Supportive Messages × Week × Time | -0.04 | 0.03 | -1.25 |  | 0.03 | 0.02 | 1.40 |  | 0.04 | 0.02 | 1.81 |  | 0.06 | 0.03 | 2.29 |
| Intervention + Supportive Messages × Week × Time | -0.01 | 0.03 | -0.38 |  | -0.01 | 0.02 | -0.57 |  | 0.00 | 0.02 | 0.06 |  | 0.03 | 0.02 | 1.47 |
| Fixed mindset | -0.03 | 0.07 | -0.44 |  | 0.14 | 0.06 | 2.09 |  | 0.05 | 0.06 | 0.88 |  | 0.06 | 0.05 | 1.17 |
| Intervention + Neutral Messages × Fixed Mindset | 0.02 | 0.08 | 0.20 |  | -0.09 | 0.07 | -1.26 |  | -0.03 | 0.06 | -0.47 |  | -0.01 | 0.06 | -0.15 |
| Control + Supportive Messages × Fixed Mindset | 0.06 | 0.10 | 0.60 |  | -0.16 | 0.09 | -1.87 |  | -0.11 | 0.08 | -1.49 |  | -0.14 | 0.07 | -2.12 |
| Intervention + Supportive Messages × Fixed Mindset | -0.03 | 0.08 | -0.42 |  | -0.08 | 0.07 | -1.08 |  | 0.00 | 0.06 | -0.01 |  | -0.04 | 0.06 | -0.67 |
| Stress-Is-Debilitating Mindset | -0.04 | 0.08 | -0.51 |  | 0.07 | 0.07 | 1.12 |  | 0.06 | 0.06 | 1.07 |  | 0.04 | 0.05 | 0.85 |
| Intervention + Neutral Messages × Stress-Is-Debilitating Mindset | -0.03 | 0.08 | -0.31 |  | 0.05 | 0.07 | 0.71 |  | 0.04 | 0.06 | 0.56 |  | 0.02 | 0.06 | 0.44 |
| Control + Supportive Messages × Stress-Is-Debilitating Mindset | 0.08 | 0.10 | 0.82 |  | 0.11 | 0.09 | 1.20 |  | 0.05 | 0.08 | 0.69 |  | -0.03 | 0.07 | -0.47 |
| Intervention + Supportive Messages × Stress-Is-Debilitating Mindset | -0.01 | 0.08 | -0.14 |  | 0.05 | 0.07 | 0.70 |  | 0.01 | 0.06 | 0.14 |  | -0.01 | 0.06 | -0.14 |
| Perceived Social Stress | -0.50 | 0.03 | -18.89 |  | 0.40 | 0.02 | 17.43 |  | 0.34 | 0.02 | 16.87 |  | 0.23 | 0.02 | 12.81 |
| Gender | 0.06 | 0.02 | 2.47 |  | 0.06 | 0.02 | 2.87 |  | 0.03 | 0.02 | 1.57 |  | -0.01 | 0.02 | -0.83 |
| URM | 0.08 | 0.02 | 3.46 |  | -0.03 | 0.02 | -1.37 |  | -0.03 | 0.02 | -1.80 |  | -0.01 | 0.02 | -0.34 |
| FG | 0.00 | 0.02 | 0.09 |  | -0.02 | 0.02 | -0.75 |  | -0.02 | 0.02 | -1.13 |  | 0.01 | 0.02 | 0.32 |

**Appendix S12. Multilevel Linear Models Showing Lack of Demographic Moderation**

We tested a secondary model for the primary outcomes that was identical to the primary multilevel linear model (see Appendix S10), except that the three demographic contrasts (gender, URM status, and FG status) were included as moderators instead of pre-intervention fixed and stress-is-debilitating mindset beliefs. There were no significant Condition × Gender, Condition × URM, or Condition × FG interactions on either of the outcomes (|*t*| < 1.09 for each interaction; see Table S5).

Table S5. *Fixed Effects from Multilevel Linear Models Testing Demographic Moderation for Primary Outcomes.*

|  | Stress Appraisals | | |  | Choice of Challenging Question | | |
| --- | --- | --- | --- | --- | --- | --- | --- |
| Term | b | SE | t |  | b | SE | t |
| (Intercept) | 3.38 | 0.07 | 51.38 |  | 0.31 | 0.03 | 9.69 |
| Intervention + Neutral Messages | 0.17 | 0.07 | 2.36 |  | 0.04 | 0.04 | 1.12 |
| Control + Supportive Messages | 0.16 | 0.09 | 1.88 |  | 0.02 | 0.04 | 0.41 |
| Intervention + Supportive Messages | 0.32 | 0.07 | 4.43 |  | 0.05 | 0.04 | 1.51 |
| Week | 0.04 | 0.02 | 1.63 |  | -0.02 | 0.01 | -1.62 |
| Intervention + Neutral Messages × Week | -0.02 | 0.03 | -0.66 |  | 0.01 | 0.01 | 0.67 |
| Control + Supportive Messages × Week | 0.05 | 0.03 | 1.41 |  | 0.02 | 0.02 | 0.97 |
| Intervention + Supportive Messages × Week | 0.02 | 0.03 | 0.74 |  | 0.03 | 0.01 | 1.82 |
| Gender | -0.04 | 0.06 | -0.62 |  | -0.03 | 0.03 | -0.83 |
| Intervention + Neutral Messages × Gender | 0.02 | 0.07 | 0.37 |  | -0.01 | 0.03 | -0.33 |
| Control + Supportive Messages × Gender | 0.03 | 0.08 | 0.41 |  | -0.01 | 0.04 | -0.33 |
| Intervention + Supportive Messages × Gender | 0.02 | 0.07 | 0.34 |  | -0.01 | 0.03 | -0.34 |
| URM | -0.04 | 0.07 | -0.50 |  | 0.00 | 0.04 | -0.07 |
| Intervention + Neutral Messages × URM | -0.01 | 0.08 | -0.13 |  | 0.00 | 0.04 | 0.07 |
| Control + Supportive Messages × URM | -0.01 | 0.09 | -0.11 |  | 0.05 | 0.05 | 1.09 |
| Intervention + Supportive Messages × URM | 0.00 | 0.08 | 0.01 |  | 0.02 | 0.04 | 0.48 |
| FG | -0.02 | 0.07 | -0.36 |  | 0.02 | 0.04 | 0.70 |
| Intervention + Neutral Messages × FG | -0.01 | 0.08 | -0.15 |  | -0.01 | 0.04 | -0.13 |
| Control + Supportive Messages × FG | 0.03 | 0.09 | 0.30 |  | -0.04 | 0.05 | -0.82 |
| Intervention + Supportive Messages × FG | 0.01 | 0.08 | 0.18 |  | -0.03 | 0.04 | -0.69 |
| Fixed mindset | -0.11 | 0.02 | -5.88 |  | -0.02 | 0.01 | -2.51 |
| Stress-Is-Debilitating Mindset | -0.22 | 0.02 | -9.84 |  | -0.01 | 0.01 | -0.92 |
| Perceived Social Stress | -0.13 | 0.02 | -6.10 |  | -0.03 | 0.01 | -2.33 |

**Appendix S13. Raw Means and Standard Deviations by Condition**

Raw means and standard deviations by intervention condition for the post-intervention manipulation check outcomes are presented in Table S6. Raw means and standard deviations by condition in the 2 × 2 design for the primary outcomes (averaged across the 10 weeks in which they were measured) are presented in Table S7.

Table S6. *Raw Means (Standard Deviations) by Intervention Condition for Post-Intervention Manipulation Check Outcomes.*

|  | Control | | |  | Intervention | | |
| --- | --- | --- | --- | --- | --- | --- | --- |
| Variable | *M* | *SD* | *N* |  | *M* | *SD* | *N* |
| Post-intervention stress-is-debilitating mindset | 3.39 | 0.83 | 314 |  | 2.77 | 0.86 | 1216 |
| Post-intervention fixed mindset | 2.50 | 1.13 | 313 |  | 2.30 | 1.11 | 1215 |

Table S7. *Raw Means (Standard Deviations) by Condition for Primary Outcomes.*

|  | Control + Neutral Messages | | |  | Intervention + Neutral Messages | | |  | Control + Supportive Messages | | |  | Intervention + Supportive Messages | | |
| --- | --- | --- | --- | --- | --- | --- | --- | --- | --- | --- | --- | --- | --- | --- | --- |
| Variable | *M* | *SD* | *N* |  | *M* | *SD* | *N* |  | *M* | *SD* | *N* |  | *M* | *SD* | *N* |
| Stress appraisals (average) | 3.40 | 0.90 | 144 |  | 3.54 | 0.87 | 668 |  | 3.49 | 0.80 | 186 |  | 3.72 | 0.82 | 640 |
| Choice of challenging question (average) | 0.33 | 0.37 | 144 |  | 0.35 | 0.39 | 668 |  | 0.32 | 0.38 | 186 |  | 0.37 | 0.39 | 640 |

**Appendix S14. Preregistration File**

The preregistration can be accessed at <https://osf.io/fchyn> and is also reproduced below.

**Study Information**

**Title:** Teachers' Provision of Affordances for the Synergistic Mindset (Intro Psych F21-S22)

**Description:** In this study, we will manipulate teachers’ communication of support (or “affordances”) for the “synergistic mindset”—the belief that intelligence is malleable and that stress can be an enhancing (rather than debilitating) factor in increasing one’s performance (Yeager et al., under review). This will be manipulated in the context of an Introductory Psychology course in a public university. Students will receive the affordance either if they got a “synergistic mindset” intervention, or not.

**Hypotheses:**

***Research Questions***

*RQ1:* Will students who receive a synergistic mindset intervention and the affordances manipulation show the most positive appraisals of stress?

*RQ2:* Will students who receive a synergistic mindset intervention and the affordances manipulation show the most positive SNS responses (skin conductance and skin temp) during a stressful quiz?

*RQ3:* Will students who receive a synergistic mindset intervention and the affordances manipulation show the most positive daily stress and wellbeing responses?

**Design Plan**

**Study type:** Experiment - A researcher randomly assigns treatments to study subjects; this includes field or lab experiments. This is also known as an intervention experiment and includes randomized controlled trials.

**Blinding:** For studies that involve human subjects, they will not know the treatment group to which they have been assigned.

Personnel who interact directly with the study subjects (either human or non-human subjects) will not be aware of the assigned treatments. (Commonly known as “double blind”)

**Study design:** This study is a 2 × 2 factorial design. Students will be randomly assigned to a synergistic mindsets condition (75%) or control condition (25%), and within these conditions, randomly assigned to either an affordance treatment condition (50%) or affordance control condition (50%). Larger proportions of students were randomized to the synergistic mindsets condition because previous studies have established the effects of the intervention, and now the focus is on the effect of affordances among those who previously received the mindset intervention.

**Randomization:** Randomization to the synergistic mindsets intervention will be done using the Qualtrics randomizer. Randomization to an affordance condition will be done by sorting participants randomly (in R) and alternately assigning them to affordance treatment or affordance control. The reason is that the affordance manipulation includes four doses (see below) and the Qualtrics randomizer would not ensure that participants are kept in the same affordance condition across doses.

**Sampling Plan**

**Existing data:** Registration prior to creation of data.

**Explanation of existing data**:

**Data collection procedures:** Data will be collected from students in an Introductory Psychology course. Students will complete either the synergistic mindsets intervention or a control activity (each short online modules) in the first two weeks of the semester. Subsequently, students will complete four brief reflection activities, either reinforcing the treatment message (affordance treatment condition) or conveying an unrelated message (affordance control condition). Throughout the semester, students will complete once-weekly ecological momentary assessments (EMAs) assessing their stress appraisals and more general experiences of stress and wellbeing. In addition, one time each week, approximately 12 students will attend class in person and provide physiological data. Specifically, these students will be outfitted with Empatica E4 wristbands (measuring skin conductance, skin temp, etc.), and they will also provide saliva samples before and after class. This will allow us to assess students’ stress responses as they go through course activities, including a potentially-stressful performance activity (e.g., a quiz).

**Sample size:** ~1,500 students providing EMA data, ~200 students providing physiological data.

**Sample size rationale:** We will collect EMA data from all students over two semesters of the course. We do not know the final sample of students who will take the class. We will collect E4 data from as many students per week as possible, given the 13 devices that our lab has access to, and given the number of weeks in the semester, and given unpredicted absences (e.g. due to COVID).

**Stopping rule:** We will collect as much data as possible over the course of two semesters: Fall 2021 and Spring 2022.

**Variables**

**Manipulated variables:** There are two experimental manipulations, crossed in a 2 × 2 factorial design (see data collection procedures above).

**Measured variables:**

***Outcomes:***

(1) Appraisals of stress (specific to the class quiz and to the course generally)

(2) Trajectory of skin conductance and skin temp reactivity “events” (i.e., striking increases in SNS reactivity) during the stressful class quiz. Events will be coded using a publicly-available algorithm that counts striking increases in SCL and ST over each several-second period.

(3) Self-reported daily internalizing symptoms (global self-evaluations, threat-type stress responses, and anxiety) when negative stress is high. Negative stress will be measured using the lab’s standard operating procedure, which is a continuous measure of the negativity rating, when participants have reported a social-evaluative stressor (see Yeager et al., 2021).

**Indices:** See “f21_synergistic_affordances_indices” file.

[f21_synergistic_affordances_indices.docx]

**Analysis Plan**

**Statistical models**

***Analytic Model:***

All statistical models will compare the focal condition—stress mindset intervention + affordances—to the pure control condition—control intervention + no affordances. Significant effects are expected for this contrast. In addition, the other two conditions will be compared to the pure control condition. We expect effects to be directionally weaker and potentially non-significant.

As a follow-up, we will directly test the effects of the affordances, by testing whether the stress mindset intervention + affordances condition is different from the stress mindset intervention + no affordances condition.

***RQ1:*** An OLS regression will test predict stress appraisals after the benchmark with three dummy variables, one for each of the non-pure-control conditions, and covariates. The primary analysis will estimate the effect of the mindset intervention + affordances condition versus the pure control.

***RQ2:*** Two mixed-effects regressions will compare the between-condition trajectories (a) skin conductance events, and (b) skin temp events, both during the stressful “benchmark” quizzes. Trajectories over time will be modeled using polynomial functional forms, or non-parametric methods (e.g. splines), depending on which method improves AIC/BIC the most. The primary analysis will compare the trajectory for the mindset intervention + affordances condition versus the trajectory for the pure control. If the data are not able to be modeled with standard regression methods, we will change to using the conservative BCF

***RQ3:*** A mixed-effect regression will predict a composite of internalizing symptoms as a function of time, stress negativity, condition, and covariates. The primary analysis will compare the simple effect of the mindset intervention + affordances condition versus the pure control, while experiencing more negative stress (i.e. +1SD on the negative stress rating scale).

**Transformations:** Survey outcomes will be untransformed.

**Inference criteria:** p < .05, two-tailed. We will also use Bayesian Causal Forest analyses as a robustness check and follow the lab’s standard operating procedures for reporting Bayesian treatment effects (e.g. ATE and CATE and 90% posterior intervals).

**Data exclusion:** Physiology data will be excluded if the sensors’ data are out of range (signaling that the sensors became disconnected from the skin). Participants in the control condition may be excluded if they also received the synergistic mindset intervention in another course on campus. This cannot be known in advance because the Qualtrics surveys are independent.

**Missing data:** Data will be imputed for covariates and moderators, if necessary. No condition variables or outcome variables will be imputed.

**Exploratory analyses:** We will also explore effects on additional survey records and administrative outcomes. We are collecting saliva samples to assess hormones and epigenetic factors related to stress and coping, but we do not currently have funding to analyze those samples, and so we cannot commit to using them as outcome variables.

**Appendix S15. CONSORT Checklist**

The CONSORT Checklist for this study is included below.

|  |  | Reporting Item | Page Number |
| --- | --- | --- | --- |
| **Title and Abstract** |  |  |  |
| Title | [#1a](https://www.goodreports.org/reporting-checklists/consort/info/#1a) | Identification as a randomized trial in the title. | 1 |
| Abstract | [#1b](https://www.goodreports.org/reporting-checklists/consort/info/#1b) | Structured summary of trial design, methods, results, and conclusions | 2 |
| **Introduction** |  |  |  |
| Background and objectives | [#2a](https://www.goodreports.org/reporting-checklists/consort/info/#2a) | Scientific background and explanation of rationale | 3-6 |
| Background and objectives | [#2b](https://www.goodreports.org/reporting-checklists/consort/info/#2b) | Specific objectives or hypothesis | 6 |
| **Methods** |  |  |  |
| Trial design | [#3a](https://www.goodreports.org/reporting-checklists/consort/info/#3a) | Description of trial design (such as parallel, factorial) including allocation ratio. | 7-9 |
| Trial design | [#3b](https://www.goodreports.org/reporting-checklists/consort/info/#3b) | Important changes to methods after trial commencement (such as eligibility criteria), with reasons | 9 |
| Participants | [#4a](https://www.goodreports.org/reporting-checklists/consort/info/#4a) | Eligibility criteria for participants | 7 |
| Participants | [#4b](https://www.goodreports.org/reporting-checklists/consort/info/#4b) | Settings and locations where the data were collected | 7 |
| Interventions | [#5](https://www.goodreports.org/reporting-checklists/consort/info/#5) | The experimental and control interventions for each group with sufficient details to allow replication, including how and when they were actually administered | 7-8 |
| Outcomes | [#6a](https://www.goodreports.org/reporting-checklists/consort/info/#6a) | Completely defined prespecified primary and secondary outcome measures, including how and when they were assessed | 9-10 |
| Sample size | [#7a](https://www.goodreports.org/reporting-checklists/consort/info/#7a) | How sample size was determined. | 6 |
| Sample size | [#7b](https://www.goodreports.org/reporting-checklists/consort/info/#7b) | When applicable, explanation of any interim analyses and stopping guidelines | SI 31 |
| Randomization - Sequence generation | [#8a](https://www.goodreports.org/reporting-checklists/consort/info/#8a) | Method used to generate the random allocation sequence. |  |
| SI 30 |  |  |  |
| Randomization - Sequence generation | [#8b](https://www.goodreports.org/reporting-checklists/consort/info/#8b) | Type of randomization; details of any restriction (such as blocking and block size) |  |
| SI 29-30 |  |  |  |
| Randomization - Allocation concealment mechanism | [#9](https://www.goodreports.org/reporting-checklists/consort/info/#9) | Mechanism used to implement the random allocation sequence (such as sequentially numbered containers), describing any steps taken to conceal the sequence until interventions were assigned | SI 30-31 |
| Randomization - Implementation | [#10](https://www.goodreports.org/reporting-checklists/consort/info/#10) | Who generated the allocation sequence, who enrolled participants, and who assigned participants to interventions | SI 31 |
| Blinding | [#11a](https://www.goodreports.org/reporting-checklists/consort/info/#11a) | If done, who was blinded after assignment to interventions (for example, participants, care providers, those assessing outcomes) and how. | 7 |
| Blinding | [#11b](https://www.goodreports.org/reporting-checklists/consort/info/#11b) | If relevant, description of the similarity of interventions | N/A |
| Statistical methods | [#12a](https://www.goodreports.org/reporting-checklists/consort/info/#12a) | Statistical methods used to compare groups for primary and secondary outcomes | 10-12 |
| Statistical methods | [#12b](https://www.goodreports.org/reporting-checklists/consort/info/#12b) | Methods for additional analyses, such as subgroup analyses and adjusted analyses | 10-12 |
| Outcomes | [#6b](https://www.goodreports.org/reporting-checklists/consort/info/#6b) | Any changes to trial outcomes after the trial commenced, with reasons | 9 |
| **Results** |  |  |  |
| Participant flow diagram (strongly recommended) | [#13a](https://www.goodreports.org/reporting-checklists/consort/info/#13a) | For each group, the numbers of participants who were randomly assigned, received intended treatment, and were analysed for the primary outcome | 31 |
| Participant flow | [#13b](https://www.goodreports.org/reporting-checklists/consort/info/#13b) | For each group, losses and exclusions after randomization, together with reason | 31 |
| Recruitment | [#14a](https://www.goodreports.org/reporting-checklists/consort/info/#14a) | Dates defining the periods of recruitment and follow-up | 7-9 |
| Recruitment | [#14b](https://www.goodreports.org/reporting-checklists/consort/info/#14b) | Why the trial ended or was stopped | SI 31 |
| Baseline data | [#15](https://www.goodreports.org/reporting-checklists/consort/info/#15) | A table showing baseline demographic and clinical characteristics for each group | SI 8 |
| Numbers analysed | [#16](https://www.goodreports.org/reporting-checklists/consort/info/#16) | For each group, number of participants (denominator) included in each analysis and whether the analysis was by original assigned groups | SI 20-21 |
| Outcomes and estimation | [#17a](https://www.goodreports.org/reporting-checklists/consort/info/#17a) | For each primary and secondary outcome, results for each group, and the estimated effect size and its precision (such as 95% confidence interval) | 12-16 |
| Outcomes and estimation | [#17b](https://www.goodreports.org/reporting-checklists/consort/info/#17b) | For binary outcomes, presentation of both absolute and relative effect sizes is recommended | N/A |
| Ancillary analyses | [#18](https://www.goodreports.org/reporting-checklists/consort/info/#18) | Results of any other analyses performed, including subgroup analyses and adjusted analyses, distinguishing pre-specified from exploratory | SI 24-28 |
| Harms | [#19](https://www.goodreports.org/reporting-checklists/consort/info/#19) | All important harms or unintended effects in each group (For specific guidance see CONSORT for harms) | N/A |
| **Discussion** |  |  |  |
| Limitations | [#20](https://www.goodreports.org/reporting-checklists/consort/info/#20) | Trial limitations, addressing sources of potential bias, imprecision, and, if relevant, multiplicity of analyses | 18-19 |
| Interpretation | [#22](https://www.goodreports.org/reporting-checklists/consort/info/#22) | Interpretation consistent with results, balancing benefits and harms, and considering other relevant evidence | 16-19 |
| Registration | [#23](https://www.goodreports.org/reporting-checklists/consort/info/#23) | Registration number and name of trial registry | 2, 10 |
| Generalisability | [#21](https://www.goodreports.org/reporting-checklists/consort/info/#21) | Generalisability (external validity, applicability) of the trial findings | 18-19 |
| **Other information** |  |  |  |
| Interpretation | [#22](https://www.goodreports.org/reporting-checklists/consort/info/#22) | Interpretation consistent with results, balancing benefits and harms, and considering other relevant evidence | 16-19 |
| Registration | [#23](https://www.goodreports.org/reporting-checklists/consort/info/#23) | Registration number and name of trial registry | 2, 10 |
| Protocol | [#24](https://www.goodreports.org/reporting-checklists/consort/info/#24) | Where the full trial protocol can be accessed, if available | 2, 10 |
| Funding | [#25](https://www.goodreports.org/reporting-checklists/consort/info/#25) | Sources of funding and other support (such as supply of drugs), role of funders | 21 |

**References**

Aronson, E. (1999). The power of self-persuasion. *American Psychologist*, *54*(11), 875–884. https://doi.org/10.1037/h0088188

Aronson, J. M., Fried, C. B., & Good, C. (2002). Reducing the effects of stereotype threat on African American college students by shaping theories of intelligence. *Journal of Experimental Social Psychology*, *38*(2), 113–125. https://doi.org/10.1006/jesp.2001.1491

Hecht, C. A., Dweck, C. S., Murphy, M. C., Kroeper, K. M., & Yeager, D. S. (in press). Efficiently exploring the causal role of contextual moderators in behavioral science. *Proceedings of the National Academy of Sciences*.

Jamieson, J. P., Crum, A., Goyer, J. P., Marotta, M. E., & Akinola, M. (2018). Optimizing stress responses with reappraisal and mindset interventions: An integrated model. *Anxiety, Stress, & Coping*, *2*, 1–17. https://doi.org/10.1080/10615806.2018.1442615

Jamieson, J. P., Mendes, W. B., & Nock, M. K. (2013). Improving acute stress responses: The power of reappraisal. *Current Directions in Psychological Science*, *22*(1), 51–56. https://doi.org/10.1177/0963721412461500

Molden, D. C., & Dweck, C. S. (2006). Finding “meaning” in psychology: A lay theories approach to self-regulation, social perception, and social development. *American Psychologist*, *61*(3), 192–203. https://doi.org/10.1037/0003-066X.61.3.192

Rege, M., Hanselman, P., Solli, I. F., Dweck, C. S., Ludvigsen, S., Bettinger, E., Crosnoe, R., Muller, C., Walton, G., & Duckworth, A. (2021). How can we inspire nations of learners? An investigation of growth mindset and challenge-seeking in two countries. *American Psychologist*, *76*(5), 755.

Yeager, D. S., Bryan, C. J., Gross, J. J., Murray, J. S., Krettek Cobb, D., HF Santos, P., Gravelding, H., Johnson, M., & Jamieson, J. P. (2022). A synergistic mindsets intervention protects adolescents from stress. *Nature*, *607*(7919), 512–520.

Yeager, D. S., & Dweck, C. S. (2020). What can be learned from growth mindset controversies? *American Psychologist*, *75*(9), 1269–1284. https://doi.org/10.1037/amp0000794

Yeager, D. S., Hanselman, P., Walton, G. M., Murray, J. S., Crosnoe, R., Muller, C., Tipton, E., Schneider, B., Hulleman, C. S., Hinojosa, C. P., Paunesku, D., Romero, C., Flint, K., Roberts, A., Trott, J., Iachan, R., Buontempo, J., Yang, S. M., Carvalho, C. M., … Dweck, C. S. (2019). A national experiment reveals where a growth mindset improves achievement. *Nature*, *573*(7774), 364–369. https://doi.org/10.1038/s41586-019-1466-y
